# Longitudinal study on immunologic, lipoproteomic, and inflammatory responses indicates the safety of sequential COVID-19 vaccination

**DOI:** 10.1007/s00109-025-02527-y

**Published:** 2025-03-12

**Authors:** Jurissa Lang, Andres Bernal, Julien Wist, Siobhon Egan, Sze How Bong, Oscar Millet, Monique Ryan, Aude-Claire Lee, Drew Hall, Philipp Nitschke, Reika Masuda, Allison Imrie, Elaine Holmes, Jeremy Nicholson, Ruey Leng Loo

**Affiliations:** 1 Australian National Phenome Centre and Centre for Computational and Systems Medicine, Health Futures Institute, Murdoch University, 5 Robin Warren Drive, Perth, WA 6150 Australia; 2 Centro de Investigación Cooperativa en Biociencias -CIC bioGUNE, Precision Medicine and Metabolism Laboratory, Basque Research and Technology Alliance, Bizkaia Science and Technology Park, 48160 Derio, Spain; 3 School of Biomedical Sciences, University of Western Australia, Nedlands, WA 6009 Australia; 4 Nutrition Research, Department of Metabolism, Nutrition and Reproduction, Faculty of Medicine, Imperial College London, Sir Alexander Fleming Building, London, SW7 2AZ UK; 5 Institute of Global Health and Innovation, Imperial College London, Faculty Building South Kensington Campus, London, SW7 2AZ UK; 6 Chemistry Department, Universidad del Valle, Cali, 76001 Colombia; 7 Department of Metabolism, Digestion and Reproduction, Faculty of Medicine, Imperial College London, Sir Alexander Fleming Building, South Kensington London, SW7 2AZ UK

**Keywords:** COVID-19 vaccine, IgG responses, Inflammation, Longitudinal study, Metabolomics, SARS-CoV-2 infection

## Abstract

**Abstract:**

COVID-19 vaccines are crucial in reducing SARS-CoV-2 transmission and severe health outcomes. Despite widespread administration, their long-term systemic effects on human metabolism remain inadequately understood. This longitudinal study aims to evaluate IgG responses, 34 cytokines, 112 lipoproteins, and 21 low-molecular-weight metabolites in 33 individuals receiving two to four COVID-19 vaccine doses. Changes in metabolic profiles for the first 16 days post each dose of vaccine, and up to 480 days post-initial dose, were compared to baseline (before vaccination). Additionally, metabolic profiles of vaccinated participants were compared to a reference cohort of unvaccinated individuals without prior exposure to SARS-CoV-2 infection (controls) and SARS-CoV-2 cases. Positive IgG responses were observed in 78.8% (*N* = 26) of participants after the first dose, reaching 100% with subsequent doses. The most common side effects were localized pain at the injection site and “flu-like” symptoms, reported by > 50% of participants. Systemic side effects, e.g., sore lymph nodes, fatigue, and brain fog, were reported but showed no significant correlations to IgG responses. Transient temporal changes were observed for cytokine IP10 (CXCL10) and glutamic acid around the third vaccine dose. Compared to the reference cohort, 497 vaccinated samples (95.0%) had profiles similar to the controls, while the remaining 26 samples with prior infection exposures were similar to mild cases of SARS-CooV-2 infection. In conclusion, COVID-19 vaccination did not induce lasting changes in inflammatory and metabolic responses, nor did it induce changes similar to mild cases of SARS-CoV-2 infection. This supports the metabolic safety of the vaccine and contributes to increased vaccine confidence.

**Key messages:**

- Minimal changes in inflammatory/metabolic markers up to 480 days post-vaccination.
- Transient increase in IP10 (CXCL10) and glutamic acid around the third dose.
- Post-vaccination IgG response did not alter metabolic profiles like SARS-CoV-2 cases.
- Our findings provide insights into the safety of repeated COVID-19 vaccinations.

**Supplementary Information:**

The online version contains supplementary material available at 10.1007/s00109-025-02527-y.

## Introduction

Since the onset of the SARS-CoV-2 pandemic, over 775 million cases have been registered, resulting in more than 7 million fatalities worldwide [1]. While most infected individuals have recovered, a significant proportion experience long-term effects lasting months to years [2]. Since the first COVID-19 vaccine became available in December 2020, over 13.6 billion doses have been administered globally with 67% of the world’s population completing a primary series [3]. The widespread deployment of vaccines has significantly reduced hospitalization and mortality and mitigated the risk of long COVID [4, 5]. Despite their efficacy, rare severe side effects such as encephalomyelitis, myocarditis, and pericarditis have been reported [6, 7]. Notably, the Oxford-AstraZeneca vaccine has been recently withdrawn from the market [8]. This highlights the need for thorough investigations into the immunological, inflammatory, and metabolic impacts of COVID-19 vaccines, both immediately post-vaccination and over the long term.

Metabolic phenotyping provides a holistic view of systemic metabolism by quantifying low molecular weight metabolites [9]. Previous research utilized this method to assess the immune response to vaccine, identifying efficacy predictors and potential mechanisms [10, 11]. In COVID-19 vaccination studies, metabolic phenotyping, either alone or in combination with other “omics” technologies such as lipidomics or proteomics, has been used to evaluate the efficacy and molecular perturbations in individuals receiving COVID-19 vaccines [12], particularly the Sinovac-CoronaVac vaccine [13–15].

This longitudinal study aimed to measure IgG levels to confirm individual responses to COVID-19 vaccination and to explore its effects on immunometabolism and metabolic pathways in healthy individuals by assessing 34 cytokines and 133 metabolic parameters within the first 16 days post each vaccination, with additional measurements up to day 480, totalling 26 time points. Comparative analyses were performed between vaccinated individuals and two reference groups: those with mild SARS-CoV-2 infection and exposed controls. This approach aimed to determine whether the vaccine induces immune responses leading to post-vaccination sequelae and/or triggers metabolic changes similar to those observed in SARS-CoV-2 infections.

## Methods

### Study cohorts

**Fig. 1:**
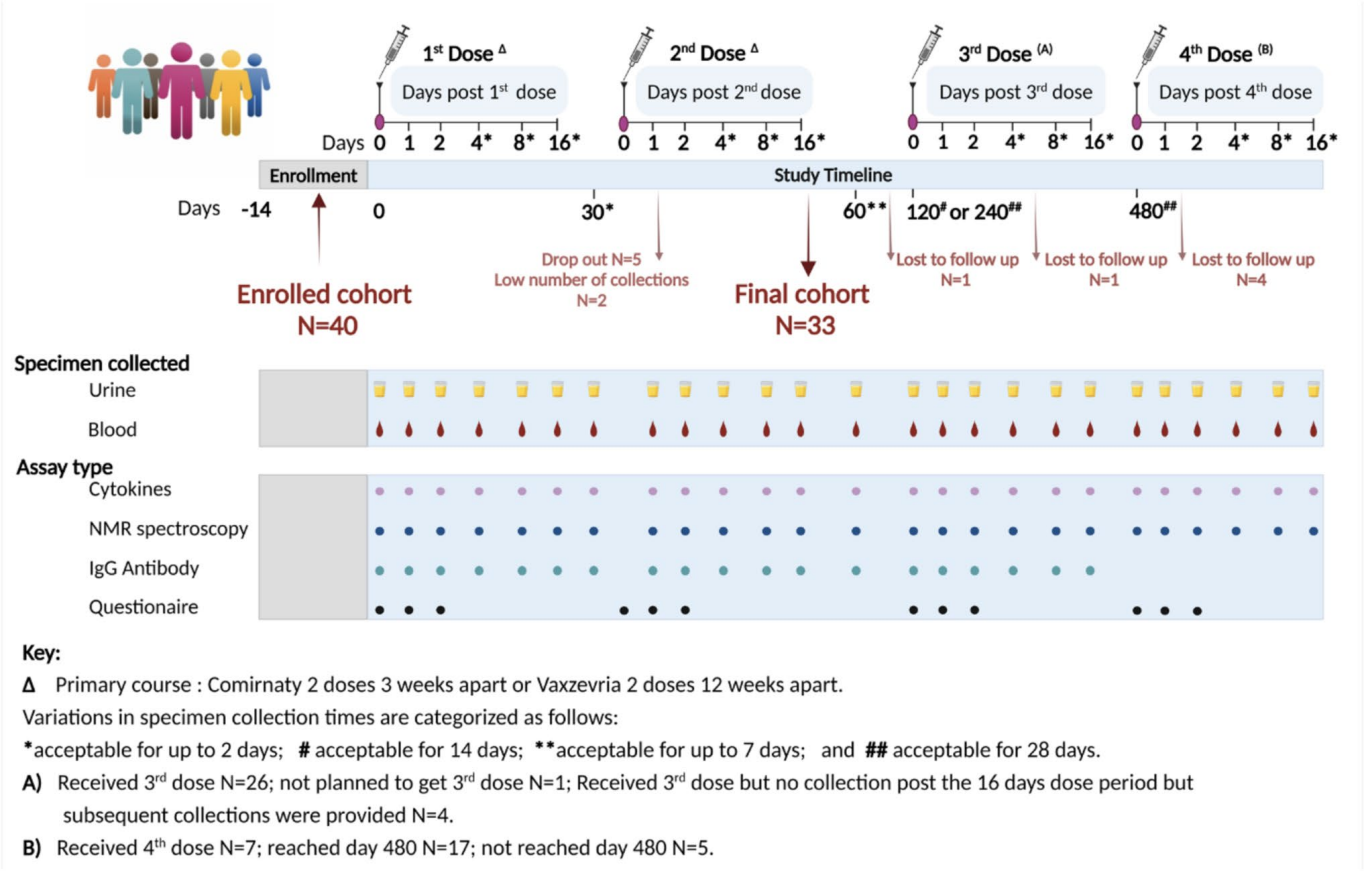
Schematic of the study design: vaccination schedule in relation to the sample collection for each time-point, together with the type of assays performed and dropout rates

Two cohorts were utilized in this study. The COVID-19 vaccine cohort, a longitudinal study, investigates the effects of repeated COVID-19 vaccinations over a 16-month period. This cohort consists of 40 healthy adults aged 18 and above from Western Australia, who received up to four doses of vaccines between June 2021 and December 2022. Participants received at least two doses of either the mRNA vaccine Comirnaty™ (Pfizer-BioNTech, BNT162b2) or the adenovirus vector vaccine Vaxzevria™ (Oxford-AstraZeneca, ChAdOx1-S-AZD1222), following the Australian Technical Advisory Group on Immunization (ATAGI) guidelines [16]. The COVID-19 vaccine cohort was applied to evaluate both the short term (days 1, 2, 4 (± 2), 8 (± 2), and 16 (± 2) post each vaccination) and long term (days 30 (± 2), 60 (± 7), 120 (± 14), 240 (± 28), and 480 (± 28) after the first dose) effects of COVID-19 vaccines on the immunometabolism and metabolic pathways. Figure 1 shows a summary of the study design together with the sample collection time points. To investigate whether similar metabolic changes were elicited in individuals who received multiple doses of the COVID-19 vaccine compared to those with SARS-CoV-2 infection, a SARS-CoV-2 reference cohort was established. This cohort included 32 individuals with mild SARS-CoV-2 infection confirmed by RT-PCR, who did not require hospitalization, and 95 control samples collected pre-pandemic from healthy individuals. Both groups matched for age range with the vaccinated cohort. All participants provided informed consent, and ethical approvals were obtained from the Murdoch University Human Research Ethics Committee (project numbers 2021/49, 2020/052, and 2020/053). Refer to the supplementary material for further information on both the COVID-19 vaccinated and SARS-CoV-2 reference cohorts.

#### Sample preparation

Fasting serum and plasma samples were collected using 10 mL silica-sprayed (BD Vacutainer® 367,895) and sodium heparin (BD Vacutainer® 367,874) tubes, respectively. Blood collection procedures were performed by qualified phlebotomists. The collected blood samples were incubated at 4 °C for a maximum of 60 min before centrifugation at 13,000* g* for 11 min at 4 °C. The resulting plasma and serum samples were aliquoted and stored at − 80 °C until analysis. A pooled quality control (PQC) sample for each matrix was prepared by pooling an equal amount (10 µL) from each sample collected up to March 2022.

### Laboratory assays and processing

- ***Anti-SARS-CoV-2 Spike 1 (S1) IgG assay***

This assay was performed on the COVID-19 vaccination cohort, which included 25 volunteers who had received up to their third vaccine dose by March 2022. The humoral response to the COVID-19 vaccine was evaluated using RecombiVirus ELISA kits for Human Anti-SARS-CoV-2 IgG (Alpha Diagnostics International, TX, USA). Samples were thawed, centrifuged, and diluted to minimize non-specific binding. Each 96-well plate contained blanks, positive controls, and calibrators. Optical density readings were obtained at 450 nm and converted to IgG concentrations (U/mL). An IgG reading above 1.2 U/mL indicated a positive response.

b) ***Cytokine analysis***

Quantitative analysis of 34 cytokines (see Supplementary Table 1 for full list) was performed on both the COVID-19 vaccination cohort and the SARS-CoV-2 reference cohort using the Cytokine & Chemokine 34-Plex Human ProcartaPlex™ Panel 1A assay kit on the MagPix detection system. Each plate contained serial dilutions of antigen standards, negative control blanks, and pooled quality control (PQC) samples. Serum samples were thawed, added to a 96-well plate with capture beads, incubated, and washed. Detection antibodies were added, followed by further incubation and washing. Median fluorescence intensity (MFI) values were processed using the “scluminex” function and data were normalized to baseline prior to data analysis.

c) ***NMR spectroscopic measurements***

NMR measurements were conducted on a Bruker 600 MHz spectrometer. Samples were thawed, centrifuged, and mixed with phosphate buffer before being transferred to NMR tubes. Three experiments were performed: a 1D experiment with solvent suppression, a T2 relaxation-filtered spin-echo experiment, and a 2D J-resolved experiment. Data were processed using Bruker Topspin. Twenty-one low-molecular-weight metabolites and 112 parameters across various lipoprotein subclasses were determined using Bruker IVDr quantification.

Refer to supplementary materials for additional details on each laboratory assay and processing procedure.

### Data analysis

Principal components analysis (PCA) [17] was conducted on 112 lipoproteins and 21 low-molecular-weight metabolites measured by NMR to identify trends and outliers. Orthogonal projection to latent structures-discriminant analysis (OPLS-DA) was employed to assess acute metabolic changes post-vaccination and longer-term impacts by comparing baseline samples with those collected at various time points post-vaccination. To compare the metabolic profiles of vaccinated individuals with those of mild SARS-CoV-2 cases or controls, OPLS-DA models discriminating SARS-CoV-2 cases from controls were constructed using the SARS-CoV-2 reference cohort, involving a Monte Carlo resampling strategy (2000 iterations). Vaccinated samples were projected onto these resampled models and considered to exhibit metabolic profiles similar to controls if classified as such in at least 1500 of the 2000 resampled OPLS-DA models. This high acceptance threshold (> 75%) aimed to minimize false positives. Data were mean-centered and scaled to unit variance before performing PCA and OPLS-DA. Each OPLS-DA model underwent sevenfold cross-validation, with significance determined by a permutation test (*N* = 100) [18]. The Q^2^Y parameter assessed model performance, and significant metabolic signatures were identified through loadings analysis followed by pairwise Kruskal–Wallis tests with Bonferroni correction (*p* < 0.05). For ease of visualization, an eruption plot was used to display metabolic signatures differentiating between groups (e.g., mild SARS-CoV-2 vs. controls), using Cliff’s Delta on the x-axis and OPLS-DA loadings on the y-axis. The metabolic signatures were color-coded based on statistical significance, with the absolute log-transformed *p*-value adjusted using for Bonferroni correction for multiple comparisons. Additionally, univariate functional principal component analysis (FPCA) with the PACE algorithm [19, 20] was utilized to model individual vaccination trajectories, focusing on cytokines indicative of inflammatory responses and significant metabolic signatures. Paired Wilcoxon tests, adjusted for multiple testing using the Benjamini–Hochberg procedure, were used to assess deviations from baseline (*p* < 0.01). Refer to supplementary material for further details.

## Results and discussion

### Cohort characteristics

A total of 33 (82.5%) participants (20 women and 13 men) remained for further analyses after two participants (5.0%) were excluded due to a low number of specimens obtained (< 8 blood specimens) following the first two doses of vaccination, and five participants (12.5%) withdrew from the study (Fig. 1). The cohort had a mean age of 36.9 years, with a standard deviation (SD) of 11.2 years. Generally, male participants were older, with a mean age of 39.9 years (range 21–65) compared to female participants, who had a mean age of 34.9 years (range 20–57). The mean body mass index (BMI) was comparable across both sexes. Overall, 63.6% (*N* = 21) of the participants were healthy with no reported comorbidities (Table 1). Of these 33 participants, 30 (90.9%) received at least two doses of the Comirnaty™ vaccine, while the remaining three individuals received two primary doses of Vaxzevria™ vaccine, followed by booster doses of the Comirnaty™ vaccine. Nine individuals (27.3%) received their third vaccine dose (first booster) around day 120, and 21 participants (63.6%) received it around day 240, while two participants (6.1%) did not receive a booster dose, and one individual (3.0%) was lost to follow-up. Seven participants received a fourth dose (second booster) approximately 480 days after their first dose of vaccination (Fig. 1). Ten individuals provided samples on day 480, at the end of the study period, while the remaining individuals have yet to reach day 480. Most missed collections occurred within the first 2 days post-vaccination, mainly due to scheduling conflicts with weekends/public holidays (*N* = 125, 18.0%) or vaccine side effects (*N* = 44, 6.3%). A total of 523 blood samples were obtained for NMR and cytokine analysis.

**Table 1:** Characteristics of participants, stratified by type of primary vaccine dose, who received up to four doses of COVID-19 vaccination from June 2021 to December 2022

	Comirnaty	Vaxzevria	Total
	(*N* = 30)	(*N* = 3)	(*N* = 33)
Age, years (SD)	35.8 (10.1)	48 (18.2)	36.9 (11.2)
Age groups, number			
< 40 years	20	1	21
> 40 years	10	2	12
Gender, number			
Female	18	2	20
Male	12	1	13
Body mass index*, mean (SD) kg/m^2^			
Normal weight	21.3 (2.1)	-	21.3 (2.1)
Overweight	26.7 (1.2)	25.1 (N/A^#^)	26.5 (1.2)
Obese	30.7 (0.5)	31.8 (0.9)	31.1 (0.8)
Comorbidities, number (%)			
Cardiovascular system	2 (6.1)	0 (0)	2 (6.1)
Central nervous system	3 (9.1)	2 (6.1)	5 (15.2)
Endocrine system	3 (9.1)	0 (0)	3 (9.1)
Gastrointestinal system	1 (3)	0 (0)	1 (3.0)
Respiratory system	1 (3)	0 (0)	1 (3.0)
Others	1 (3)	0 (0)	1 (3.0)
None	20 (60.6)	1 (3)	21 (63.6)
Vaccine status, number			
3 doses	23	3	26
4 doses	5	2	7

Key: *Missing data from 3 Comirnaty participants, ^#^comprised of one participant

### Increased anti-SARS-CoV-2 Spike 1 IgG humoral response post COVID-19 vaccination

**Fig. 2:**
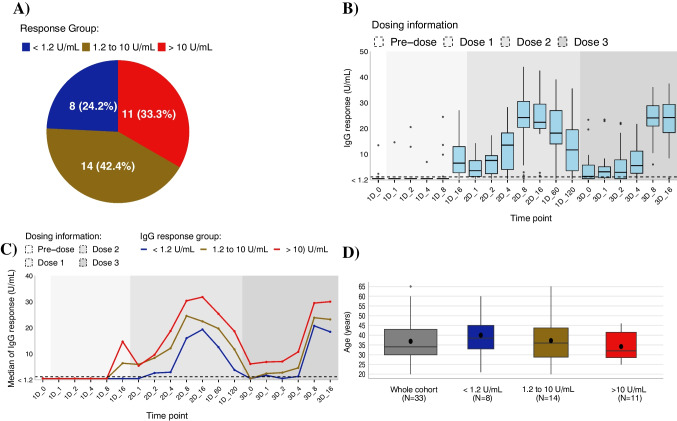
IgG response stratified by IgG response groups or dosing information. **A** Pie charts of COVID-19 vaccine cohort (*N* = 33), stratified by IgG response group; **B** box plot of IgG responses for all participants, with the horizontal dashed line at 1.2 U/mL indicating a positive response to COVID-19 vaccine. The x-axis indicates the number of doses and days post-vaccination. The first two characters represent the dose, and the number, separated by the dashed line, represents the number of days post-dose. For example, “1D_4” represents day 4 post first dose of vaccine administration; **C** median IgG trajectory response up to the third dose of vaccination is shown, with the x-axis indicating time points as described in **B**; and **D** box plots showing the median (horizontal line), mean (black dot), and interquartile range of age for the whole cohort, and stratified by IgG response groups

Individual IgG response trajectories are shown in Supplementary Fig. 1. At baseline, 32 participants (97.0%) showed no IgG response (< 1.2 U/mL) and one participant (VAC22C) displayed positive IgG results. After the first vaccine dose, 8 participants (24.2%) showed no IgG response, while 26 (78.8%) exhibited positive responses. Among these, 14 (42.4%) had IgG levels between 1.2 and 10 U/mL, and 11 (33.3%) had levels > 10 U/mL (Fig. 2A). Following the second dose, all participants had positive IgG responses, confirming vaccine efficacy (Fig. 2B). IgG levels remained above 1.2 U/mL before the third dose at day 120, except for one individual who received Vaxzevria™. However, 40% of those who received their booster at day 240 had IgG levels < 1.2 U/mL before the booster. Participants who exhibited no IgG response (< 1.2 U/mL) or had IgG response between 1.2 and 10 U/mL consistently showed lower IgG response compared to those with IgG responses exceeding 10 U/mL prior to receiving the second dose of vaccination (Fig. 2C). Younger participants tended to have higher IgG responses, with mean ages decreasing as IgG levels increased: 40.0 years (SD 13.0) for < 1.2 U/mL, 37.2 years (SD 12.7) for 1.2–10 U/mL, and 34.2 years (SD 7.6) for > 10 U/mL (Fig. 2D). This aligns with studies showing a decline in IgG response in older age groups or those with comorbidities [21–23].

**Fig. 3:**
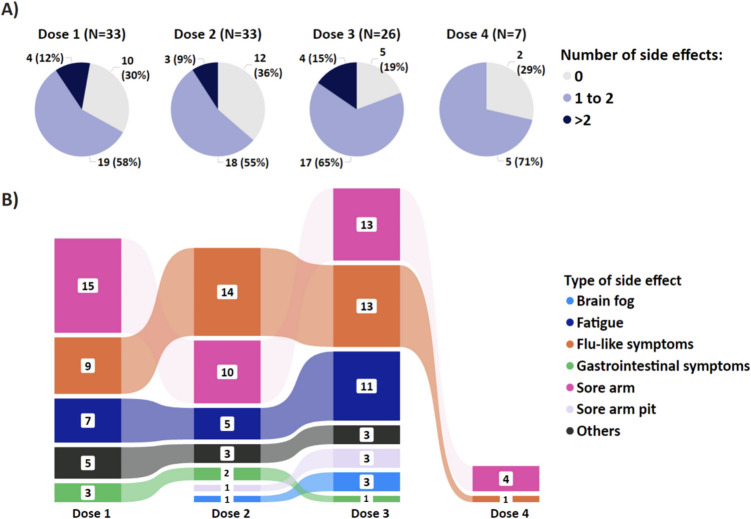
Self-reported side effects. **A** Number of self-reported side-effects for all participants after each successive vaccination; and **B** the ribbon plot illustrates the changes in the ranking of self-reported side effect, stratified by each successive vaccination

The proportion of participants reporting no side effects for each successive vaccination were 30.3% (*N* = 10), 36.4% (*N* = 12), 19.2% (*N* = 5), and 28.6% (*N* = 2) (Fig. 3A). Side effects were typically reported within 3 days post-vaccination, with sore arm and flu-like symptoms being most common. Other side effects included fatigue and gastrointestinal symptoms. Brain fog and sore armpit were reported after subsequent vaccinations, consistent with existing literature [24–27]. As previously reported, serious side effects such as interstitial lung disease, myocarditis/pericarditis, transverse myelitis, and encephalomyelitis [6, 28, 29] were not observed in this study. Overall, 27.3%, 57.6%, 56.4%, and 16.7% of participants reported systemic side effects after one, two, three, and four doses, respectively (Fig. 3B). Participants with IgG levels < 1.2 U/mL reported fewer side effects compared to those with > 10 U/mL for the first two doses, consistent with a previous study showing association between systemic symptoms and greater antibody responses [30]. This trend was not evident for the third dose, with average number of side effects per participant being 2.2, 2.3, and 2.4 for < 1.2 U/mL, 1.2–10 U/mL, and > 10 U/mL, respectively. No specific trends were discerned for the fourth dose due to limited data (Supplementary Fig. 2).

### Chemokine IP10 (CXCL10) exhibits transient temporal fluctuations post vaccination

**Fig. 4:**
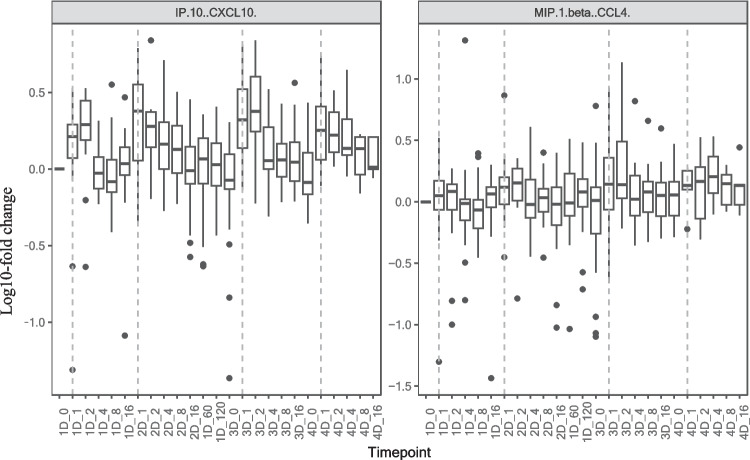
The boxplots illustrate the median fluctuations of IP-10 (CXCL10) and MIP-1 (CCL4) from baseline up to 480 days. The x-axis indicates the number of doses and days post-vaccination. The first two characters represent the dose, and the number, separated by an underscore, represents the days post-dosing. For example, “1D_4” represents day 4 post first dose of vaccine administration. The dashed vertical lines indicate one day post vaccination

PCA of 34 cytokines revealed significant inter-individual variations due to a few individuals with elevated cytokine levels even before vaccination (data not shown). One individual consistently showed increased levels of TNF-α, IL-4, IL-6, IL-13, IFN-γ, and GM-CSF, while another showed elevated levels of GRO-α, TNF-β, IL-8, IL-9, IL-23, and IL-31. Given that these differences represent real biological variation between individuals, they were preserved for subsequent FPCA analyses to explore the interplay between the temporal profiles of inflammatory markers at an individual level over a 480-day period following initial vaccination. Overall, univariate FPCA indicated minimal temporal fluctuations in cytokine levels, with narrow interquartile ranges around the median functions, while outliers influenced the 2.5th and 97.5th percentiles (Supplementary Fig. 3). Notably, IP10 (CXCL10) and MIP-1b (CCL4) showed immediate increases post-vaccination, subsiding to baseline before subsequent doses (Fig. 4). IP10 (CXCL10) plays a crucial role in immune response and is implicated in various pathological states, including the cytokine storm in SARS-CoV-2 infection [31–33]. Elevated IP10 (CXCL10) levels are associated with severe SARS-CoV-2 infection, and its reduction corresponds to patient recovery [33]. In this vaccinated cohort, despite the significant increase in IP10 (CXCL10), levels remained within the reported ranges of variation observed previously in healthy controls [33], suggesting these temporal fluctuations are unlikely to hold biological significance. This indicates that COVID-19 vaccination does not provoke inflammatory responses comparable to those observed in viral infections, highlighting the difference in systemic immunological responses elicited by the vaccine compared to SARS-CoV-2 infection.

### Absence of SARS-CoV-2-like metabolic changes in COVID-19 vaccine recipients

**Fig. 5:**
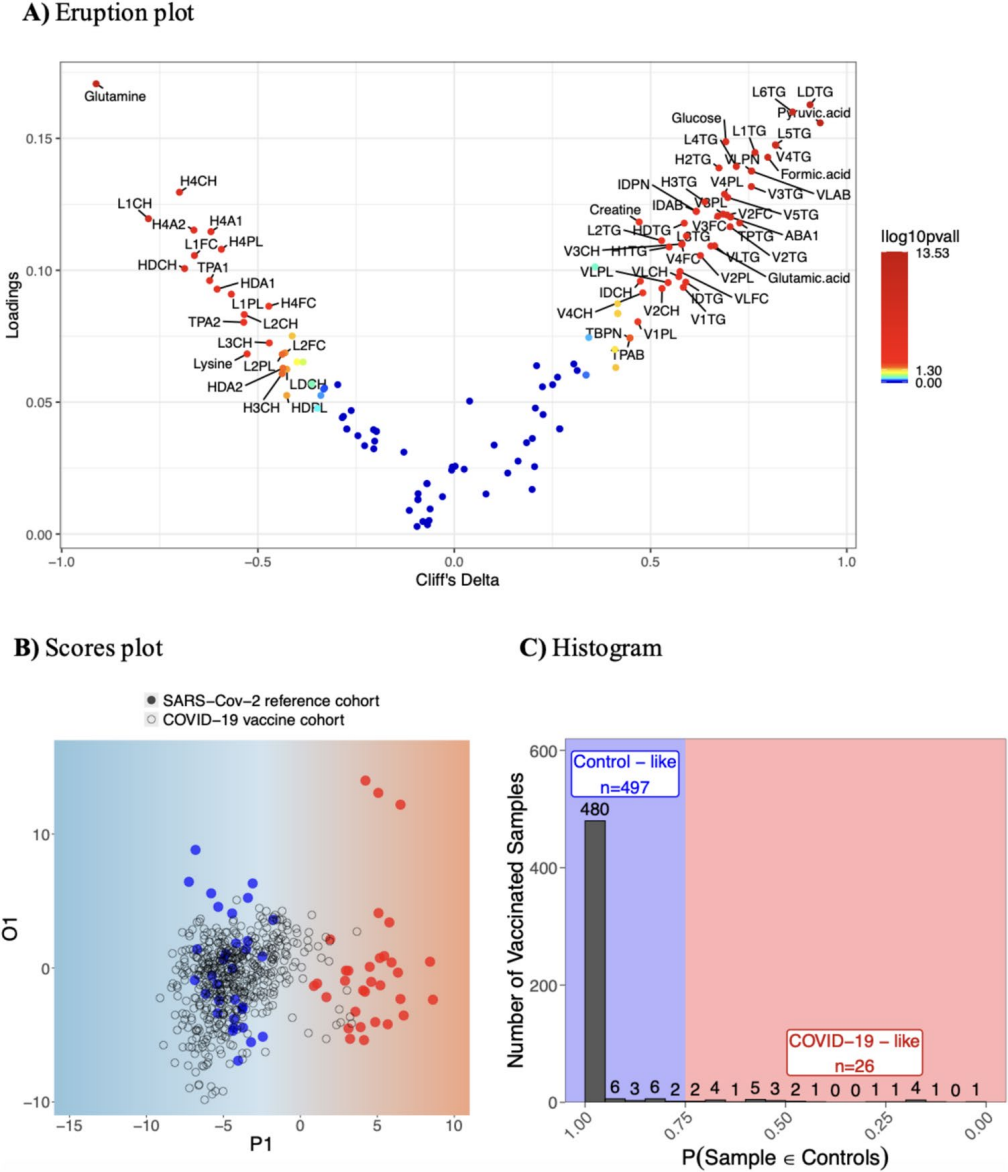
Evaluation of the impact of COVID-19 vaccine cohort, compared to mild cases of SARS-CoV-2 infection and controls, using a supervised OPLS-DA technique. **A** The eruption plot combines lipoprotein data and small molecule metabolites to differentiate mild SARS-CoV-2 cases from controls, using Cliff’s delta (abscissa) and OPLS-DA loadings (ordinate). Variables are color-coded based on Kruskal–Wallis *p*-values adjusted for Bonferroni correction for multiple comparisons. Please see Supplementary Table 2 for full list of abbreviation. **B** The scores plot represents one of the resampled OPLS-DA models for controls and mild cases of SARS-CoV-2 infection from the SARS-CoV-2 reference cohort, depicted as blue closed circles and red closed circles, respectively. The COVID-19 vaccine samples (represented as open circles) are projected onto the scores plot indicating the resemblance of the samples to either controls or mild cases of SARS-CoV-2. **C** The probability that vaccinated samples (*N* = 523), collected from 33 individuals (up to 4 doses), exhibit metabolic profiles similar to those of mild SARS-CoV-2 infection cases (represented by red panel) or healthy controls (represented by blue panel). Using the 75% similarity threshold as the cut-off point for controls, the blue panel denotes the number of vaccinated participants samples that are categorized as possessing metabolic profiles similar to the controls. Conversely, the red panel represents the number of vaccinated samples that are classified as having metabolic profiles resembling those of mild SARS-CoV-2 infections

The SARS-CoV-2 reference cohort was utilized to determine whether the activation of the immune system elicited by the COVID-19 vaccine subsequently provokes metabolic alterations comparable to those observed in mild cases of SARS-CoV-2 infections. PCA of 112 lipoproteins and 21 small molecules from the COVID-19 vaccine cohort showed tight clustering of PQC samples (Supplementary Fig. 4(A)) and no observable pattern based on IgG response group, sex, or vaccine type (data not shown), indicating these factors did not significantly influence metabolic profiles. Co-clustering of samples in the PCA suggested that individual variations in metabolic profiles outweighed vaccination effects (Supplementary Fig. 4(B)). PCA of the SARS-CoV-2 reference cohort revealed a tendency for mild cases of SARS-CoV-2 infection to co-cluster (Supplementary Fig. 4(C)), while the OPLS-DA model (R^2^Y = 89.8%, Q^2^Y = 84.9%, and p(Q^2^Y) < 0.01) showed two distinct metabolic clusters (Supplementary Figs. 4(D) to (F)). Mild SARS-CoV-2 cases exhibited elevated triglycerides (for VLDL, LDL, and HDL subfractions), glutamic acid, pyruvic acid, glucose, and formic acid. In contrast, the control group had higher levels of free cholesterol, cholesterol, HDL and LDL subfractions of phospholipids, apolipoproteins A1, glutamine, and lysine (Fig. 5A). These findings align with previous observations [34–36].

The projection of post-COVID-19 vaccinated samples onto OPLS-DA models, based on 2000 class-balanced resampling of the SARS-CoV-2 reference cohort (Fig. 5B), showed that 76.3% (*N* = 399) were consistently classified as controls across all 2000 models. Additionally, 91.8% (*N* = 480) were projected as controls by > 95% of the models (Fig. 5C). Overall, 95.0% (*N* = 497) of the samples were classified as controls in > 1500 of the 2000 OPLS-DA models, meeting the criterion for discarding the occurrence of a metabolic response similar to SARS-Cov-2 infection. The remaining 5.0% (26 samples) came from eight volunteers, with 14 samples from one volunteer (VAC203) who reported a COVID-19 diagnosis post-Europe trip and quarantine. These 14 samples spanned from the first to the third vaccine dose, during which time the volunteer was diagnosed with long COVID. Another three samples were from VAC22C, who showed a raised IgG response from baseline (Supplementary Fig. 1), suggesting prior asymptomatic SARS-CoV-2 infection rather than cross-reactivity to other coronaviruses. Four samples from VAC1D2, post-first vaccine dose, exhibited mild SARS-CoV-2-like responses in 29.6–46.3% of models. The only sample collected on day 8 post-first dose was classified as a control, marginally outside the criterion for being a mild SARS-CoV-2 case (23.2% of the resampled OPLS-DA models). Given the model’s success in identifying VAC203 and probably VAC22C as cases of SARS-CoV-2 infection, it is plausible that VAC1D2 may have been exposed to the virus around the same time as the first dose of vaccination (Supplementary Fig. 1) as no SARS-CoV-2-like perturbations were observed in subsequent doses. The remaining five samples, each from different volunteers, showed isolated mild SARS-CoV-2-like profiles. For example, a sample from VAC255, collected on day 4 post-second booster, showed a mild SARS-CoV-2-like profile in 99.9% of models. However, samples collected on days 8 and 16 post-vaccination were consistently classified as controls, while the pre-dose collection sample taken immediately prior to the fourth dose of vaccine demonstrated only a 0.2% similarity to the mild SARS-CoV-2 profile. Considering that many SARS-CoV-2 like samples were projected at the borderline between the mild cases of SARS-CoV-2 and control groups, as illustrated in one of the resampled OPLS-DA scores plot (Fig. 5B), it is plausible these five isolated samples were naïve to SARS-CoV-2, and unlikely to hold significant biological implications.

### Negligible metabolic alterations post COVID-19 vaccination with temporal glutamic acid variability

No significant OPLS-DA models differentiated the baseline samples (t = 0) from days 1, 2, 4, 8, and 16 post each dose of vaccination (20 models in total), indicating no significant metabolic changes within 2 weeks post-vaccination. Long-term impact comparisons (days 60, 120, 240, and 480) also showed no significant models, except for day 240 (R^2^Y = 0.472, Q^2^Y = 0.258, p[Q^2^Y] = 0.01) (Supplementary Fig. 5(A)), which showed higher glutamic acid levels at day 240 contrasted with higher baseline citric acid levels (Supplementary Fig. 5(B)). At day 240, 27.3% (*N* = 9) had received their first booster dose 3 months prior; the rest were yet to be vaccinated. To further investigate whether vaccine timing contributed to the differentiation between baseline and day 240, two additional OPLS-DA models were constructed, focusing on IgG response levels. These models compared non-responders (IgG < 1.2 U/mL, *N* = 8) to responders (IgG > 1.2 U/mL, *N* = 14) and non-responders to strong responders (IgG > 10 U/mL, *N* = 11). Neither model yielded significant results, suggesting IgG response levels did not contribute to the separation at baseline and day 240.

**Fig. 6:**
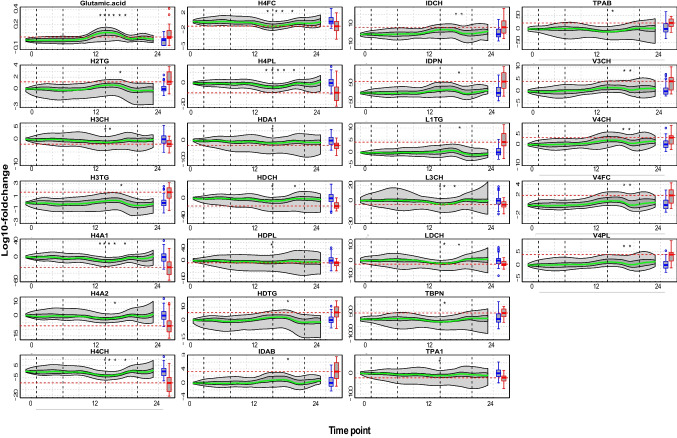
Functional boxplots, derived from FPCA, depict significant temporal fluctuations in 25 lipoproteins and glutamic acid over a period of 480 days. The green line denotes the functional median, the dark gray area represents the interquartile range, and the light gray indicates the range covered by the curves within the 2.5th and 97.5th percentiles. Vertical lines mark the time points 1 day post each vaccine dose. The boxplots alongside the functional boxplots indicate the metabolite distributions for healthy controls (blue) and mild SARS-CoV-2 infection cases (red) within reference cohort. The horizontal red line marks the median value for the mild SARS-CoV-2 infection cases. Keys for time points: 0, baseline; 1 to 5, days 1, 2, 4, 8, and 16 following the first vaccine dose; 6 to 10, days 1, 2, 4, 8, and 16 following the second vaccine dose; 11, day 60 post-first vaccine dose; 12, day 120 post-first vaccine dose; 13 to 18, days 0, 1, 2, 4, 8, and 16 following the third vaccine dose; and 19 to 24, days 0, 1, 2, 4, 8, and 16 following the fourth vaccine dose. The asterisk (*) indicates the time points that showed significantly different levels compared to baseline (Wilcoxon *p* < 0.01), after adjusting for multiple testing (Benjamini-Hochberg)

FPCA on 60 lipoprotein parameters and 7 small molecule metabolites, associated with the separation between mild SARS-CoV-2 infection and controls in the reference cohort, revealed significant deviations in 26 metabolites post-third vaccine dose (Fig. 6 and Supplementary Fig. 6). Notably, several lipoproteins, including total plasma and HDL apolipoprotein-A1 (TPA1 and HDA1), HDL subclass 4 free cholesterol (H4FC), HDL-subclass phospholipids (HDPL), cholesterols from HDL-subclass 3 (H3CH), VLDL-subclass 4 (V4CH), LDL (LDCH), and its subclass 3 (L3CH), along with glutamic acid, mirrored the fluctuations seen in mild SARS-CoV-2 cases. However, these fluctuations were transient and small compared with the perturbations observed for mild SARS-CoV-2 cases from the reference cohort (Fig. 6). Notably, glutamic acid significantly increased around the third vaccine dose, surpassing the median change for SARS-CoV-2 cases. Glutamine, metabolized into glutamate and ammonia by mitochondrial enzyme glutaminase [37], is linked to immune dysfunction, infection risk, vascular disease, and inflammation [37–41]. Studies show an inverse correlation between plasma glutamine/glutamate ratio and cardiometabolic risk factors, including blood pressure, triglyceride levels, and insulin sensitivity [41, 42]. Low glutamine levels correlate with SARS-CoV-2 severity [37, 43, 44], implicating its role in inflammation, immune dysfunction, coagulopathy, vascular occlusion, and multi-organ failure [36]. In the vaccinated cohort, the glutamine-to-glutamic acid ratio remained similar to controls (mean ratio of 8.9 for the vaccinated cohort vs 9.5 for controls in the SARS-CoV-2 reference cohort) and higher than mild SARS-CoV-2 cases (mean ratio of 4.2 in the SARS-CoV-2 reference cohort). This suggests the increase in glutamic acid alone may not adversely affect biological functions.

This study is the first to investigate both short- and long-term systemic metabolic responses to COVID-19 vaccination. Previous metabolic studies focused on vaccine effects in SARS-CoV-2 symptoms [45], mRNA vaccine responses [12], and specific cohorts like immunosuppressed patients [46] and pregnant women [47]. Here, FPCA summarized complex longitudinal data, capturing temporal changes not apparent at the population level. This provided a deeper understanding of inflammatory and metabolic response dynamics over time and with cumulative vaccinations. Comparing temporal changes in inflammatory and metabolic responses in the COVID-19 vaccine cohort with those who had mild infections or no prior exposure, it enabled the evaluation of the biological implications of these fluctuations.

It is important to recognize the limitations of this study, including the small number of participants, which may constrain broad applicability. However, multiple longitudinal sampling over 480 days partially mitigates this. The results offer valuable insights for public health policy and vaccination strategies. Firstly, the robust immune response supports continued vaccine deployment to control COVID-19 spread and reduce morbidity. This underscores the importance of re-vaccination to achieve immunity, especially as vaccine uptake has declined recently [48]. Secondly, it enriches research on long-term immunological and metabolic responses to COVID-19 vaccination over a year and multiple doses, as over 95% of samples showed healthy control-like profiles, with the remaining 5% mostly due to infection exposure. Thirdly, positive IgG responses and minimal metabolic/cytokine effects reinforce vaccine safety and efficacy, addressing vaccine hesitancy and promoting public confidence in vaccination programs. Future research with larger, diverse populations is recommended.

In conclusion, this longitudinal study demonstrates sustained immune efficacy and stability in metabolic and inflammatory markers post repeated COVID-19 vaccination. The positive IgG responses affirm the vaccine’s capacity to provoke a robust immune reaction, while subtle and transient fluctuations in inflammatory and metabolic responses indicate a favorable safety profile. These findings underscore the stability of the metabolic response despite repeated vaccinations, offering reassurance that vaccine-induced immunity is distinct from SARS-CoV-2 infection.

## Supplementary Information

Below is the link to the electronic supplementary material.

Supplementary file1 See Supplementary Tables 1 and 2 for full list of abbreviation for cytokines and lipoproteins. (PDF 2.22 MB)

## Data Availability

All data used and/or analyzed during the current study may be available from the corresponding author on reasonable request.

## Abbreviations

*1D** NMR*: 1-Dimenional Nuclear magnetic resonance spectroscopy
*ATAGI*: Australian Technical Advisory Group on Immunisation
*BMI*: Mean body mass index
*COVID-19*: Coronavirus disease 2019
*ELISA*: Enzyme-linked immunosorbent assay
*FPCA*: Functional principal component analysis
*HDL*: High-density lipoprotein
*IDL*: Intermediate-density lipoprotein
*IgG*: Immunoglobulin G
*IVDr*: In vitro diagnostics research
*LDL*: Low-density lipoprotein
*MFI*: Median fluorescence intensity
*mRNA*: Messenger ribonucleic acid
*OPLS-DA*: Orthogonal projection to latent structures discriminant analysis
*PCA*: Principal component analysis
*PQC*: Pooled quality control
*RT-PCR*: Reverse transcription-polymerase chain reaction
*SD*: Standard deviation
*SARS-CoV-2*: Severe acute respiratory syndrome coronavirus 2
*VLDL*: Very low-density lipoprotein
*TNF-α*: Tumor necrosis factor alpha
*IL-4*: Interleukin-4
*IL-6*: Interleukin-6
*IL-13*: Interleukin-13
*IFN-γ*: Interferon gamma
*GMCSF*: Granulocyte-macrophage colony-stimulating factor
*GRO-α*: Growth related oncogene alpha
*TNF-β*: Tumor necrosis factor beta
*IL-8*: Interleukin-8
*IL-9*: Interleukin-9
*IL-23*: Interleukin-23
*IL-31*: Interleukin-31
*CXCL1*: C-X-C motif chemokine ligand-1
*IL-1RA*: Interleukin-1 receptor antagonist
*IP-10*: Interferon gamma-induced protein-10
*SDF-1-α*: Stromal cell-derived factor-1 alpha
*IL-8*: Interleukin-8
*TNF-α*: Tumor necrosis factor alpha
*MIP-1β*: Macrophage inflammatory protein-1 beta
*MCP-1*: Monocyte chemoattractant protein-1
