# Supplementary material for: Longitudinal study on immunologic, lipoproteomic, and inflammatory responses indicates the safety of sequential COVID-19 vaccination
Source: J Mol Med (Berl). 2025 Mar 12;103(4):421–33. doi: 10.1007/s00109-025-02527-y (PMC12003606; doi:10.1007/s00109-025-02527-y)
Supplement: Supplementary file 1 — Supplementary file1 See Supplementary Tables 1 and 2 for full list of abbreviation for cytokines and lipoproteins. (PDF 2.22 MB) [file 109_2025_2527_MOESM1_ESM.pdf]

Longitudinal Study on Immunologic, Lipoproteomic, and Inflammatory Responses Indicates the Safety of Sequential COVID-19 Vaccination

Jurissa Lang[1], Andres Bernal[1], Julien Wist[1], Siobhan Egan[1], Sze How Bong[1], Oscar Millet[2], Monique Ryan[1], Aude-Claire Morillon[1], Drew Hall[1], Philipp Nitschke[1], Reika Masuda[1], Allison Imrie[3], Elaine Holmes[1,4], Jeremy Nicholson[1, 4,5*] and Ruey Leng Loo[1*]

1. Australian National Phenome Centre and Centre for Computational and Systems Medicine, Health Futures Institute, Murdoch University, 5 Robin Warren Drive, Perth, WA 6150, Australia
2. Centro de Investigación Cooperativa en Biociencias -CIC bioGUNE, Precision Medicine and Metabolism Laboratory, Basque Research and Technology Alliance, Bizkaia Science and Technology Park, 48160 Derio, Spain
3. School of Biomedical Sciences, University of Western Australia, Nedlands, WA 6009, Australia
4. Nutrition Research, Department of Metabolism, Nutrition and Reproduction, Faculty of Medicine, Imperial College London, Sir Alexander Fleming Building, London SW7 2AZ, United Kingdom
5. Institute of Global Health and Innovation, Imperial College London, Faculty Building South Kensington Campus, London, SW7 2AZ, United Kingdom

Supplementary Information

Cohorts

a) ***Covid-19 vaccine cohort:*** This is a multi-dose study involving repeated injection of widely used Covid-19 vaccines, with effects being monitored for up to 16 months. The study cohort comprised of 40 healthy adults aged 18 and above, residing in Western Australia (WA), who voluntarily received up to four doses of the COVID-19 vaccine between June 2021 and December 2022. All participants received at least two doses of vaccine, either with the mRNA vaccine Comirnaty™ (Pfizer-BioNTech, BNT162b2) or the adenovirus vector vaccine Vaxzevria™ (Oxford-AstraZeneca, ChAdOx1-S-AZD1222), as part of the WA Government COVID-19 vaccination program, following the recommendations of the Australian Technical Advisory Group on Immunization (ATAGI) [1]. Exclusion criteria included individuals with current acute or chronic infection, undergoing active chemotherapy or immunomodulatory therapies, diagnosed with autoimmune conditions, and undergoing active treatment; and those who had received any vaccination in the last two weeks prior to their COVID-19 vaccine.

   Mid-stream urine, fasting plasma and serum samples were collected from participants at baseline (t=0) and on days 1, 2, 4 (±2), 8 (±2) and 16 (±2) post each dose of COVID-19 vaccine. Additional urine and blood collections were performed on days 30 (±2), 60 (±7), 120 (±14), 240 (±28) and 480 (±28) after the first dose. Participants provided information on a questionnaire consisting of any previous COVID-19 test results during the pandemic. Additionally, participants recorded any post-vaccination side effects after each dose of vaccine. A schematic of the blood and urine collection schedule, along with the types of assays performed, the number of volunteers receiving each dose of the vaccine, and the dropout rates, is depicted in **Figure 1.** All participants provided written informed consent.

Participants who provided at least 8 out of the scheduled 12 specimens within the first two vaccine doses and up to day 60 from the initial vaccine dose were included. This study was approved by the Murdoch University Human Research Ethics Committee (project number 2021/49).

b) ***SARS-CoV-2 reference cohort:*** It is known that SARS-CoV-2 infection strongly influences the levels of lipoproteins and small molecules in blood samples and that these effects can persist for months [2-5]. To investigate whether similar changes in these signatures were also elicited in individuals who have received multiple doses of the COVID-19 vaccine and demonstrated a positive IgG response, a reference cohort was compiled. This reference cohort included confirmed cases of SARS-CoV-2 infection, ascertained from upper and/or lower respiratory tract swabs using reverse transcription-polymerase chain reaction (RT-PCR). Only samples from individuals who exhibited mild cases of SARS-CoV-2 infection, not requiring hospitalization for oxygen supplementation and mechanical ventilation, were included. Samples from severe SARS-CoV-2 infection requiring hospitalization were excluded from this reference cohort. Severe infections are known to cause complex metabolic changes and necessitate various medical interventions, rendering comparisons of the metabolic profiles of these individuals with those who have received the COVID-19 vaccine unsuitable. A total of 32 samples from acute mild SARS-CoV-2 patients were collected by the Basque Biobank for Research (BIOEF) and the Western Australian South Metropolitan Health Service catchment as part of the International Severe Acute Respiratory and Emerging Infection Consortium (ISARIC)/World Health Organization (WHO) pandemic trial framework (South Metropolitan Health Service Research governance office PRN:3976). The control samples from this reference cohort were collected before the COVID-19 pandemic by Osarten

Kooperativa Elkartea from an apparently healthy population (employees of the Mondragon Cooperative [Basque Country]) during their annual medical tests [2]. This control cohort thus represents a “normal” population. Both the control (N=95) and SARS-CoV-2 infected (N=32) groups were matched for age range with the COVID-19 vaccination cohort. All participants provided informed consent for clinical investigations in accordance with the Declaration of Helsinki. The sample-handling protocol was evaluated and approved by the Comitéde Ética de Investigacióncon medicamentos de Euskadi (PI+CES-BIOEF 2020-04 and PI219130). Research was conducted in accordance with the Murdoch University Human Ethics Committee approval and all data were anonymized (no. 2020/052 and 2020/053). Samples supplied by BIOEF were imported under Import Permit 0004275122 issued by the Australian Government’s Department of Agriculture, Water, and the Environment and approved by the Ministry of Health of the Spanish Government. All samples were stored at -80 °C until analysis.

Laboratory Assays and Processing

a) ***Anti-SARS-CoV-2 Spike 1 (S1) IgG Assay:*** The humoral response to the vaccine was assessed using the RecombiVirus series of ELISA kits for Human Anti-SARS-CoV-2 IgG (Alpha Diagnostics International, TX, USA) [6]. This evaluation was conducted on all serum samples collected until March 2022, of which 25 volunteers had received their third vaccine dose. In brief, serum samples were thawed at 4 °C for a maximum of 60 minutes and then centrifuged at 13,000 *g*. Subsequently, 5 µL of serum samples were diluted 1:100 with low non-specific binding sample dilutant to minimize non-specific binding and other matrix effects. Each 96-well plate included two "blanks" using sample diluent, two anti-SARS-CoV-2 S1 as positive controls, and two sets of anti-SARS-CoV-2 S1 calibrators at 1, 2.5, 5 and 10 U/mL. Automated washing steps were performed using the BIORAD BIO-

Plex Pro™ wash station. Optical density (OD) readings and raw data calculations were obtained using the Agilent BioTek Gen5™ 3.10 software on the 800™ TS absorbance reader (BioTek Instruments, Vermont, USA) at a wavelength of 450 nm. To determine the IgG concentration (U/mL) in each sample, the OD absorbance reading was converted using the average of the two standard curves generated using the manufacturer's calibrators OD reading, after deducting the average reading based on both blank samples. The absorbance reading recorded using Gen5 3.10 software was then converted to U/mL. An IgG reading exceeding 1.2 U/mL was considered a positive response to vaccination, as per the manufacturer's protocol. Within-plate reproducibility was assessed using the PQC, showing a coefficient of variation (CV) of <10% for all the plates, indicating excellent reproducibility and reliability of the results. The processed IgG data were checked for trends across different time points, relative to baseline (t=0).

b) ***Cytokines Assay:*** Quantitative analysis of 34 cytokines were measured using the Cytokine & Chemokine 34-Plex Human ProcartaPlex™ Panel 1A assay kit (Invitrogen™, Thermo Fisher Scientific International, MA, USA) on the MagPix detection system (Luminex, USA) according to manufacturer's recommendations [3]. This assay covered a spectrum of cytokines and chemokines [7] selected based on a previous study comparing SARS-CoV-2 infection with healthy controls [3]. The complete analyte panel list, along with the details of each analyte's sensitivity and standard curve range is outlined in **Supplementary Table 1**. Briefly, each plate included two sets of seven 4-fold serial dilutions of antigen standard, two negative control blanks, and a minimum of three PQC samples [3]. Serum samples were thawed at 4 °C for maximum 60 minutes, followed by the addition of 25 µL of serum to a 96-well plate containing the capture bead. The plate was then incubated overnight at 4 °C before being washed three times with the wash buffer using the magnetic

separator Bio-Plex Pro™ wash station (Bio-Rad Laboratories). Detection antibody mixes containing Streptavidin-Phycoerythrin were added to each well and incubated for 30 minutes at 20 °C on a shaker, followed by three additional washes with the wash buffer. Subsequently, an assay buffer was added and incubated for a further 5 minutes before analysis using a MagPix™ detection system. Median fluorescence intensity (MFI) values obtained from the Luminex™ multiplex immunoassay were processed using the 'scluminex' function in the 'drc' package. This process includes removing noise and eliminating background signals while considering the standards and background MFI, followed by normalization, and data filtering [8, 9]. To ensure the accuracy and reliability of the data, the reproducibility of each analyte on every plate was ensured by using a PQC sample. This PQC sample was reanalyzed at a minimum of three different intervals during the analysis of each plate. Analytes exhibiting a CV < 20% were included for further analysis. All analyses were performed using statistical software R studio using the Multiplex Immunoassays Data Analysis *drLumi* (v0.12), *drc* (v 3.0-1) packages [8]. To account for potential batch effects between plates, the library 'limma' was utilized on log-10 transformed data. The processed cytokines data for each time point were normalized to baseline (t=0).

c) ***Quantitative NMR-measured analytes:*** NMR measurements were performed on a Bruker 600 MHz Avance III HD spectrometer equipped with a 5 mm BBI probe and fitted with a Bruker SampleJet™ robot with the cooling system set to 5 °C. Calibration of the spectrometer consisted of temperature calibration with 99.8% deuterated methanol set for 310 K and water suppression with a standard 2nM sucrose sample (0.5 mL sodium trimethylsilyl propionate-[2,2,3,3–2H_4] (TSP), 2 mM NaN_3 in 10% D_2O: 90% H_2O) [10] followed by the Bruker standard Quantref sample validation and calibration, unique to the

spectrometer. [10] All experiments were completed using the Bruker *in vitro* diagnostics research (IVDr) methods [11]. A validated sampling preparation and handling protocol for blood samples was used as previously described [12]. Briefly, prior to NMR analysis, frozen samples were thawed at 4 °C for maximum 60 min and centrifuged at 13,000 *g* for 10 min at 4 °C. An equal sample volume of 350 μL of serum was mixed with 350 μL phosphate buffer (75 mM Na_2HPO_4, 2 mM NaN_3, 4.6 mM TSP in D_2O, pH 7.4 ± 0.1) and 600 μL of the resulting mixture was transferred to a SampleJet NMR tube (5 mm outer diameter). For quality control samples, a PQC sample was used. Three experiments were completed in automation with a total analysis time of 12.5 min: a ^{1}H one-dimensional (1D) experiment with solvent suppression (32 scans, relaxation delay of 4s, 96K data points, spectral width of 30 ppm, line broadening of 0.3 H*z*, zero-filled to 128K), a T_2 relaxation-filtered Carr–Purcell–Meiboom–Gill spin-echo experiment (32 scans, 72K data points, the spectral width of 20 ppm, line broadening of 0.3 H*z*, zero-filled to 128K); and a two-dimensional *J*-resolved experiment (2 scans, 40 t_1 increments, and a spectral width of 16 ppm in F1 and 78 Hz in F2). All data were processed in automation using Bruker Topspin 3.6.2 and ICON NMR to achieve phasing and baseline correction.

Lipoprotein reports consisting of 112 lipoprotein parameters for each sample were generated using the Bruker IVDr Lipoprotein Subclass Analysis (B.I.LISA) method [11], **Supplementary Table 2**. This was completed by quantifying the complex $-CH_2$ ($\delta = 1.25$) and $-CH_3$ ($\delta = 0.8$) spectral profiles of the 1D NMR spectra after normalization to the Bruker QuantRef manager within Topspin using a PLS-2 regression model. The lipoprotein subclasses included different molecular components of very low-density lipoprotein (VLDL, 0.950–1.006 kg/L), low-density lipoprotein (LDL, density 1.09–1.63 kg/L), intermediate-density lipoprotein (IDL, density 1.006–1.019 kg/L), and high-density lipoprotein (HDL, density 1.063–1.210 kg/L). The LDL subfraction was further divided

into six density classes (LDL-1 1.019–1.031 kg/L, LDL-2 1.031–1.034 kg/L, LDL-3 1.034–1.037 kg/L, LDL-4 1.037–1.040 kg/L, LDL-5 1.040–1.044 kg/L, and LDL-6 1.044–1.063 kg/L), and the HDL subfractions were divided into four different density classes (HDL-1 1.063–1.100 kg/L, HDL-2 1.100–1.125 kg/L, HDL-3 1.125–1.175 kg/L, and HDL-4 1.175–1.210 kg/L). The concentrations of 21 low-molecular-weight metabolites were determined using Bruker IVDr quantification in plasma/serum B.I.Quant-PS, after excluding those that below the limits of detection. The quantified metabolites included acetic acid, acetone, citric acid, creatine, creatinine, formic acid, glucose, glutamic acid, glutamine, glycine, histidine, D-3-hydroxybutyric acid, isoleucine, lactic acid, leucine, lysine, methionine, phenylalanine, pyruvic acid, tyrosine, and valine.

Data Analysis

Principal components Analysis (PCA) [13] was performed on the 133 analytes measured by NMR, which comprised 112 lipoproteins and 21 low-molecular-weight metabolites, to provide an overview of the data, discern trends, and identify outliers. Orthogonal Projection to Latent Structures-Discriminant Analysis (OPLS-DA) modelling was used to evaluate the multivariate metabolic response to the vaccine. All data were mean-centred and scaled to unit variance prior to PCA and OPLS-DA. Specifically, each OPLS-DA model underwent seven-fold cross-validation to assess performance and to optimize the number of orthogonal components [14]. The significance of each model was determined by a permutation test (N=100). The performance of the OPLS-DA model was ascertained through the Q^2Y parameter. The contributory metabolic signatures that distinguished between groups in the significant OPLS-DA models were identified through loadings analysis and pairwise Kruskal-Wallis tests, using the Bonferroni correction, with a significance threshold set at $p < 0.05$.

To evaluate the resemblance of the metabolic profiles of vaccinated samples to those of mild cases of SARS-CoV-2 infection, OPLS-DA models were constructed using the SARS-CoV-2 reference cohort. This incorporated a Monte Carlo resampling strategy involving 2,000 iterations. For each iteration, a class-balanced OPLS-DA model was constructed, comprising all mild cases of SARS-CoV-2 infection (N=32) and a random selection of 32 controls. This approach mitigated bias arising from unbalanced classes within the SARS-CoV-2 reference cohort. Then, vaccinated samples were projected onto each of these OPLS-DA models. The fraction of models that projected the sample into the controls class was used to determine whether the sample was more akin to a control (positive) or to a SARS-CoV-2 case (negative). To minimize false positives, a high threshold of acceptance of >75% was adopted. This threshold assigns a sample as a control if and only if it is classified as such in at least 1,500 of the 2,000 resampled OPLS-DA models. Additional OPLS-DA models were constructed to assess acute changes in the metabolic profile after each dose of the COVID-19 vaccine. OPLS-DA models compared samples collected at baseline (before vaccination, t=0) with those collected on days 1, 2, 4, 8 and 16 after each dose, in a pairwise manner. To evaluate the longer-term impact of vaccination on metabolic profiles, baseline samples were also compared with those collected on days 60, 120, 240 and 480 post-vaccination. Univariate Functional Principal Component Analysis (FPCA) with the PACE algorithm [15, 16] was employed to characterize both inter-individual and intra-individual variability and to estimate individual vaccination trajectories. Briefly, FPCA modelled individual parameter trajectories using a mean function and a covariance function. These trajectories were expressed as a linear combination of orthogonal functions, weighted by individual-specific scores [15, 16]. This analysis was performed on all cytokines indicative of inflammatory responses, and only on significant metabolic signatures that differentiated the control group from the SARS-CoV-2 infection group within the reference cohort. The bandwidth for smoothing of the covariance function

was set to 2. A functional boxplot for each analyte, based on the function scores, was created to enable the visualization of trends and variations over time. Paired Wilcoxon tests, adjusted for multiple testing using the false discovery rate, were performed to assess the significance of deviations from the baseline, with a significance level set at $p < 0.01$. All analyses were performed in R using in-house scripts along with packages "fdapace" [17] and "ropls" [18].

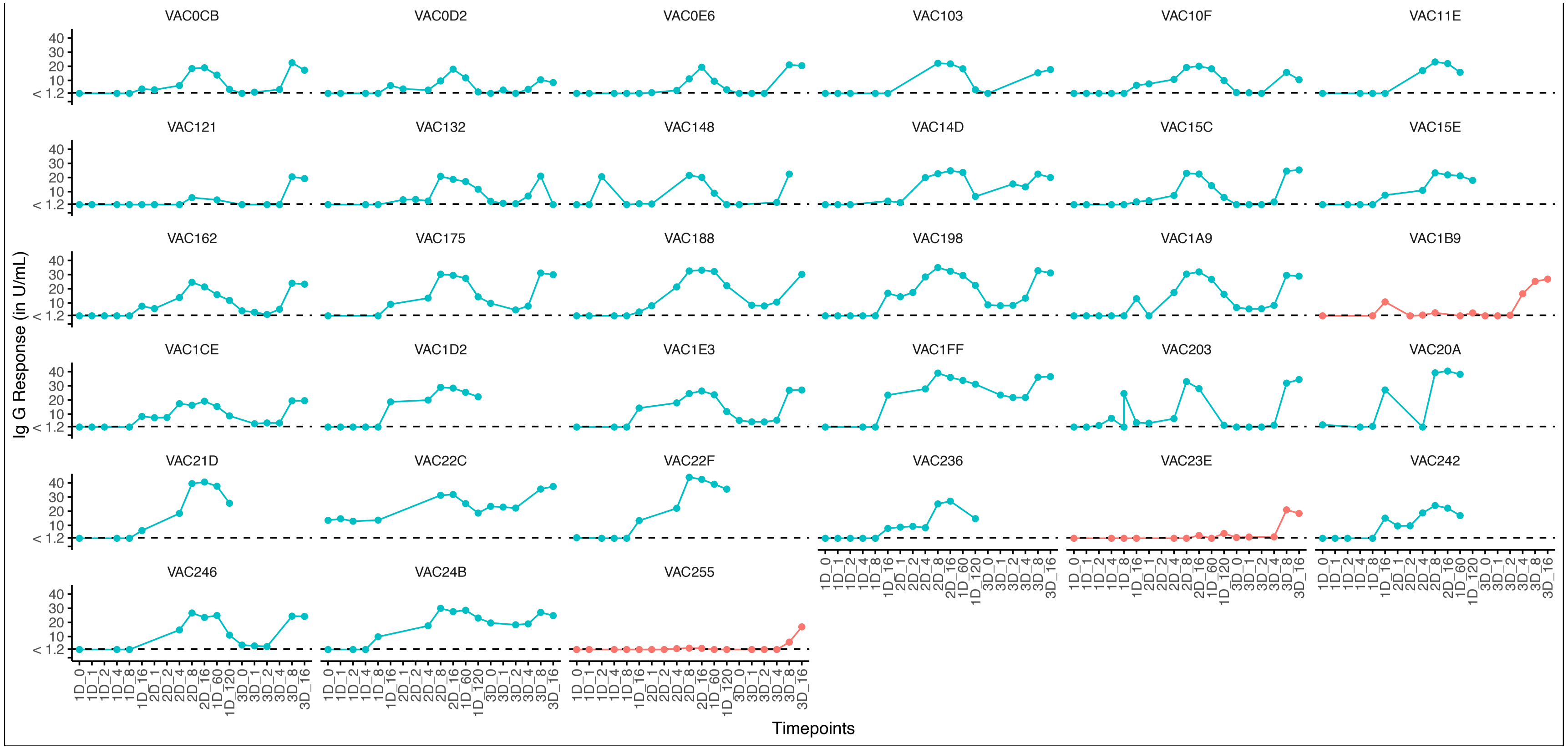

Supplementary Figure 1: IgG response trajectories over time for each participant. Individuals were color-coded according to the type of vaccine they received: red for Vaxzevria™ and blue for Comirnaty™. The x-axis indicates the number of doses and days post-vaccination. The first two characters represent the dose, and the numerical number, separated by the dash line, represents the days post-dosing. For example, '1D_4' represents day 4 post first dose of vaccine administration. The horizontal line at 1.2 U/mL indicates the threshold for IgG response.

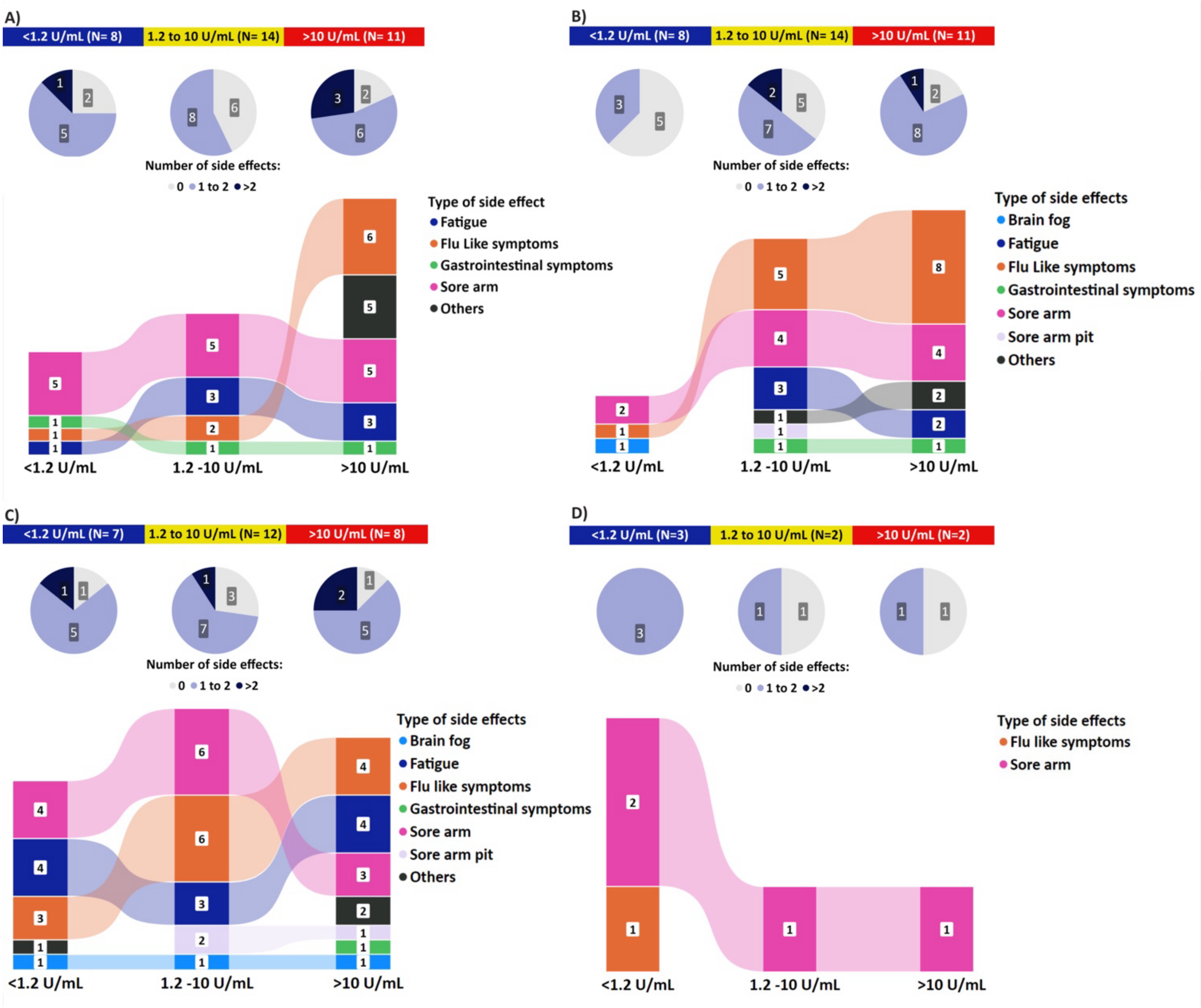

Supplementary Figure 2: **Self-reported side-effects, stratified by IgG response groups.** The ribbon plot illustrates the changes in the ranking of self-reported side-effect, stratified by IgG response groups. Accompanying pie charts display the count of reported side effects for each response group following the (A) first; (B) second; (C) third; and (D) fourth dose of vaccine.

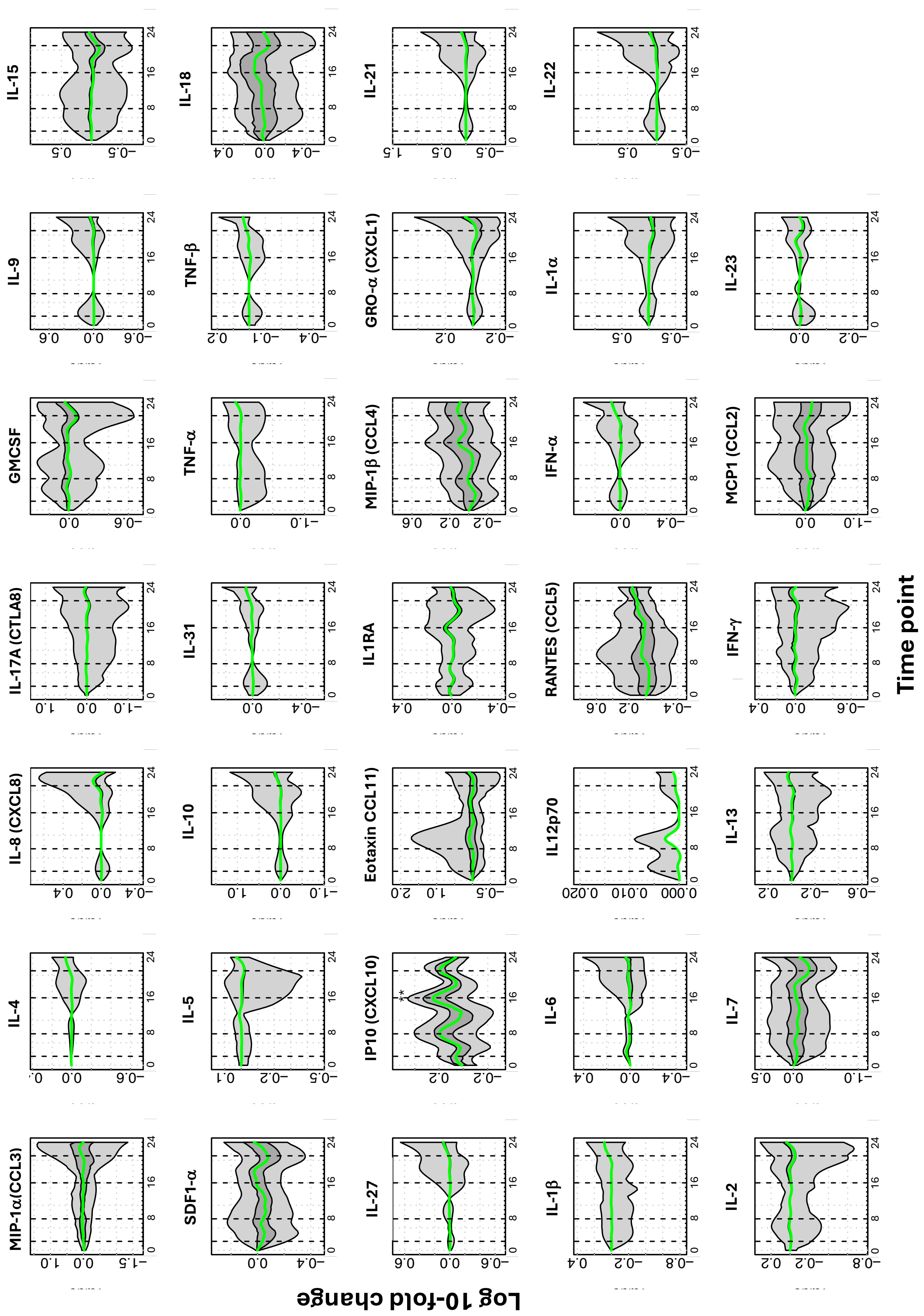
MIP-1α(CCL3)
IL-4
IL-8 (CXCL8)
IL-17A (CTLA8)
GMCSF
IL-9
IL-15
SDF1-α
IL-5
IL-10
IL-31
TNF-α
TNF-β
IL-18
IL-27
IP10 (CXCL10)
Eotaxin CCL11
IL1RA
MIP-1β (CCL4)
GRO-α (CXCL1)
IL-21
IL-1β
IL-6
IL12p70
RANTES (CCL5)
IFN-α
IL-1α
IL-22
IL-2
IL-7
IL-13
IFN-γ
MCP1 (CCL2)
IL-23
Log 10-fold change
Time point

Supplementary Figure 3: Functional boxplots, derived from FPCA, illustrate the temporal fluctuations of each chemokine from baseline (t=0) over a period of 480 days. The green line represents the functional median, while the dark gray area corresponds to the interquartile range and the light gray represents the range spanned by the curves within the 2.5th and 97.5-th percentiles. Vertical lines mark the time points one day after each dose of vaccine. Keys for time points: 0: Baseline; 1 to 5: Days 1, 2,4, 8 and 16 following the first vaccine dose; 6 to 10: Days 1, 2,4, 8 and 16 following the second vaccine dose; 11: Day 60 post-first vaccine dose; 12: Day 120 post-first vaccine dose; 13 to 18: Days 0, 1, 2, 4, 8, and 16 following the third vaccine dose; and 19 to 24: Days 0, 1, 2, 4, 8, and 16 following the fourth vaccine dose. The asterisks (*) on the first and second days after the third vaccine dose indicate these time points showed significantly higher levels of IP10 (CXCL10) compared to baseline, after adjusting for the false discovery rate at 0.01.

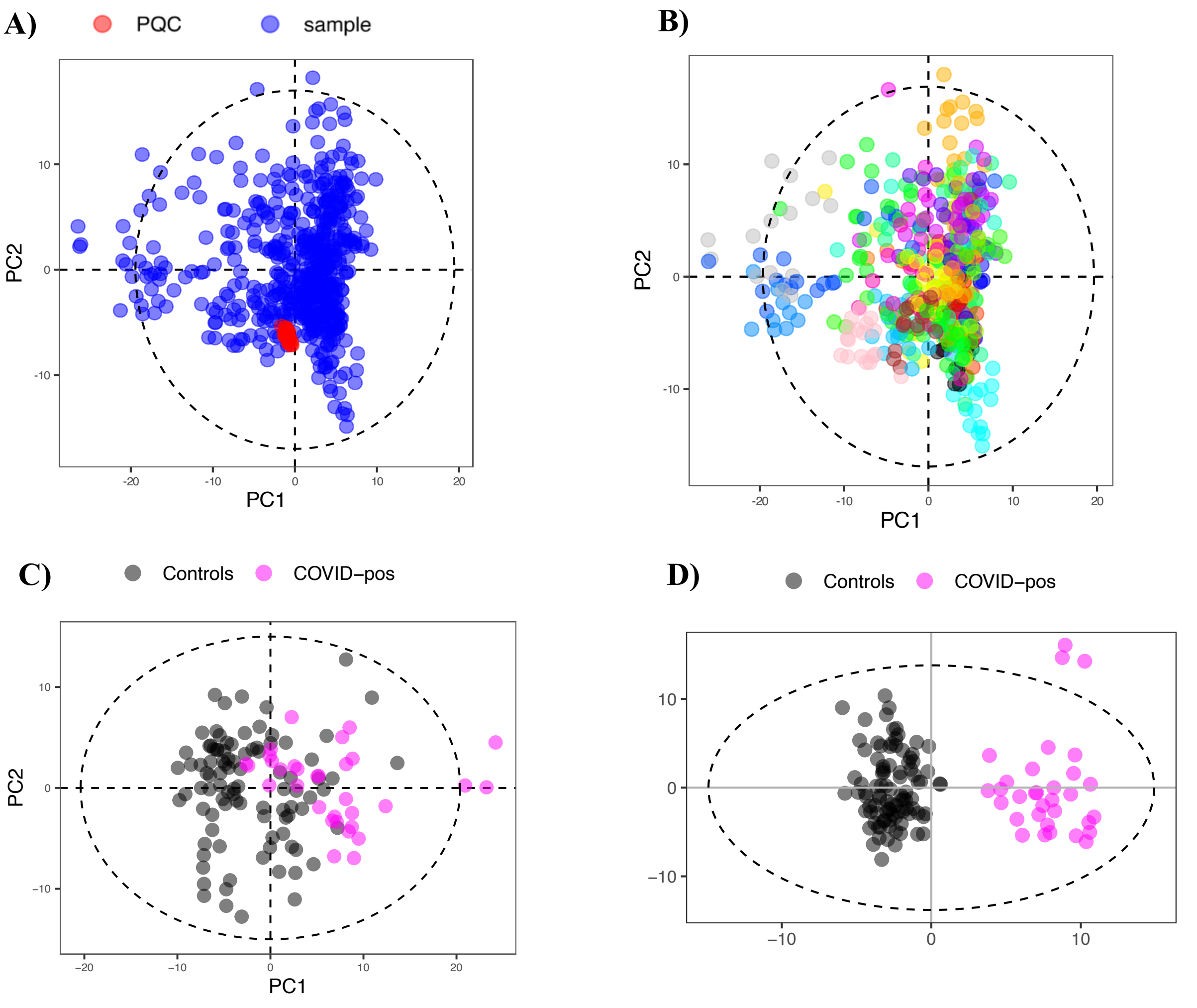

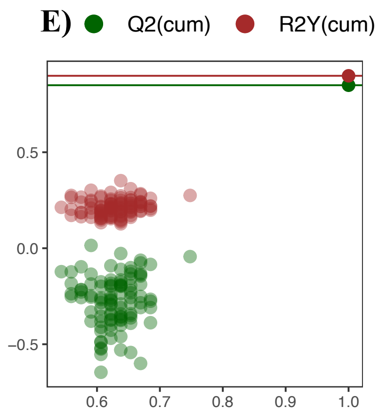

F)

Summary of OPLS-DA model statistics for SARS-CoV-2 compared to controls

Model	R^2X	R^2X (cummulative)	R^2Y	R^2Y (cum)	Q^2 Y	Q^2 Y (cummulative)
Predictive	0.174	0.174	0.575	0.575	0.557	0.557
Orthogonal component 1	0.227	0.401	0.164	0.164	0.145	0.145
Orthogonal component 2	0.0779	0.479	0.107	0.272	0.0881	0.233
Orthogonal component 3	0.1	0.579	0.0305	0.302	0.0374	0.27
Orthogonal component 4	0.108	0.687	0.0208	0.323	0.0225	0.293
**Sum**	**NA**	**0.687**	**NA**	**0.898**	**NA**	**0.849**

Supplementary Figure 4: Modelling of the COVID-19 vaccine cohort and SARS-CoV-2 reference cohort. **(A)** The PCA scores plot of the COVID-19 vaccine cohort shows a tight clustering of the pooled quality control (PQC) samples, depicted in red, which were analyzed repeatedly throughout the analytical batch, contrasting with the large variability of the samples (in blue). **(B)** The PCA scores plot of the COVID-19 vaccine cohort, without the PQC samples but color-coded by individual participants, indicates that samples from the same individuals tend to co-cluster. **(C)** The PCA scores plot of the SARS-CoV-2 reference dataset reveals a tendency for samples from mild cases of SARS-CoV-2 infection (depicted in pink) to co-cluster separately from controls (depicted in black), particularly along PC 1 and 3. **(D)** OPLS-DA model of the SARS-CoV-2 reference cohort, showing clear clustering between controls and mild cases of SARS-CoV-2 infection. **(E)** A random permutation of the OPLS-DA model was constructed, based on 100 iterations, showing the high robustness of the model. **(F)** The statistics for the OPLS-DA model based on the SARS-CoV-2 reference dataset.

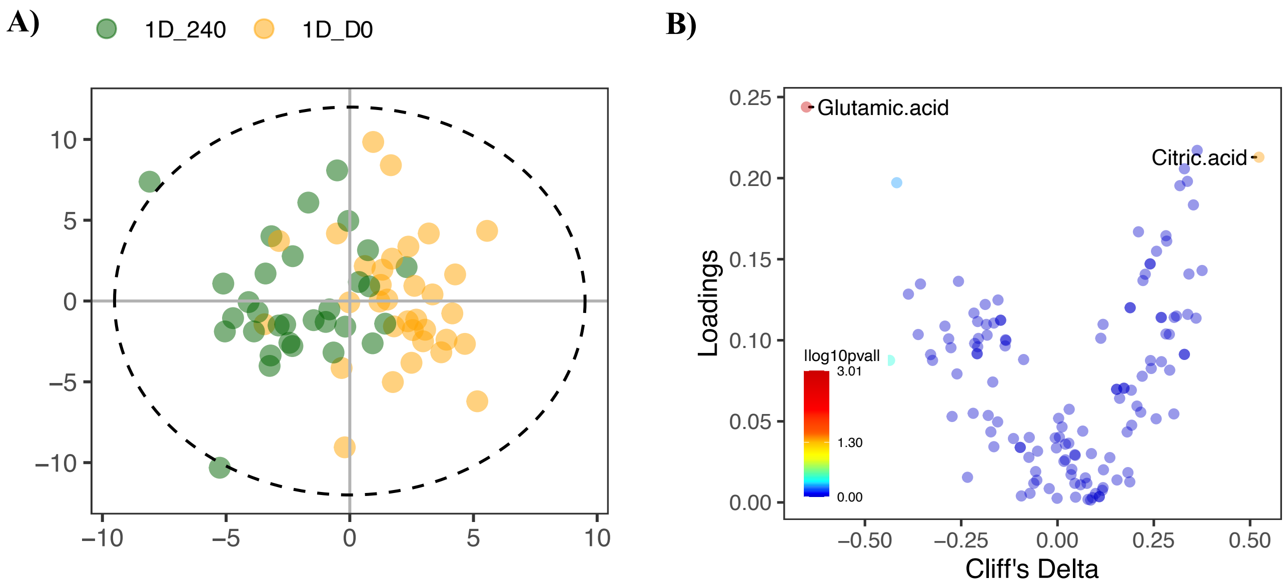

Supplementary Figure 5: OPLS-DA scores plot for the COVID-19 vaccination cohort. (A) The OPLS-DA scores plot depicts the separation between samples collected at baseline (t=0), represented in green, and samples collected at day 240, represented in yellow. (B) The OPLS-DA eruption plot reveals that a significant contributing factor to the separation between t =0 and t=240 are the elevated levels of citric acid and glutamic acid, respectively.

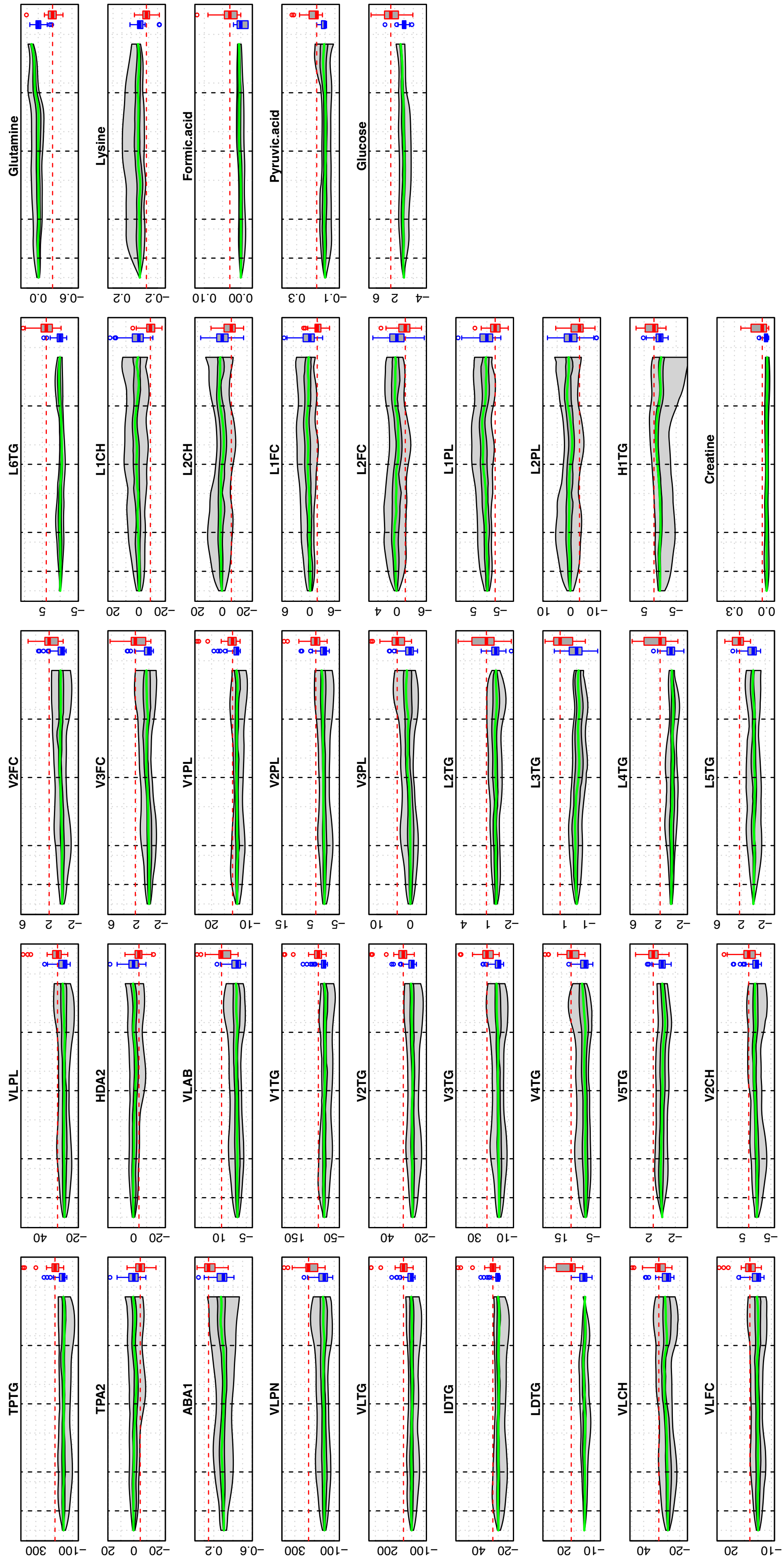
TPTG
TPA2
ABA1
VLPN
VLTG
IDTG
LDTG
VLCH
VLFC
VLPL
HDA2
VLAB
V1TG
V2TG
V3TG
V4TG
V5TG
V2CH
V2FC
V3FC
V1PL
V2PL
V3PL
L2TG
L3TG
L4TG
L5TG
L6TG
L1CH
L2CH
L1FC
L2FC
L1PL
L2PL
H1TG
Creatine
Glutamine
Lysine
Formic.acid
Pyruvic.acid
Glucose

Supplementary Figure 6: Functional boxplots, derived from FPCA, illustrate insignificant temporal fluctuations of 35 lipoproteins and 6 small molecule metabolites over a period of 480 days. The green line represents the functional median, while the dark gray area corresponds to the interquartile range and the light gray represents the range spanned by the curves within the 2.5th and 97.5-th percentiles. Vertical lines mark the time points one day subsequent to each dose of vaccine. The boxplots indicate the distributions of the corresponding analyte for healthy controls (blue) and mild cases of SARS-CoV-2 infection (red) in the SARS-CoV-2 reference cohort. The horizontal red line marks the median value for the mild SARS-CoV-2 infection. Keys for time points: 0: Baseline; 1 to 5: Days 1, 2,4, 8 and 16 following the first vaccine dose; 6 to 10: Days 1, 2,4, 8 and 16 following the second vaccine dose; 11: Day 60 post-first vaccine dose; 12: Day 120 post-first vaccine dose; 13 to 18: Days 0, 1, 2, 4, 8, and 16 following the third vaccine dose; and 19 to 24: Days 0, 1, 2, 4, 8, and 16 following the fourth vaccine dose.

Supplementary Table 1: Sensitivity and standard curve range for each cytokine and chemokine measured in this study.

Cytokine/chemokine	Abbreviation	Sensitivity (pg/mL)	Standard Curve Range (pg/mL)
Eotaxin	Eotaxin (CCL11)	1.4	0.61-2500
Granulocyte-macrophage colony-stimulating factor	GM-SCF	1.2	17.09-70000
Growth related oncogene alpha	GRO-α	2.8	2.44-10000
Interferon alpha	IFN-α	0.2	0.61-2500
Interferon gamma	IFN-γ	0.2	12.21-50000
Interleukin-1 receptor antagonist	IL-1RA	17.8	34.18-140000
Interleukin -1alpha	IL-1α	0.1	0.61-2500
Interleukin -1 beta	IL-1β	0.2	2.44-10000
Interleukin -2	IL-2	0.8	4.88-20000
Interleukin -4	IL-4	1.5	12.21-50000
Interleukin -5	IL-5	0.3	7.32-30000
Interleukin -6	IL-6	0.4	9.77-40000
Interleukin -7	IL-7	0.2	0.61-2500
Interleukin -8	IL-8 (CXCL8)	1.2	2.44-10000
Interleukin -9	IL-9	0.5	9.77-40000
Interleukin -10	IL-10	0.1	2.44-10000
Interleukin -12p70	IL12p70	0.04	6.84-28000
Interleukin -13	IL-13	0.1	2.44-10000
Interleukin -15	IL-15	1.1	3.05-12500
Interleukin -17A	IL-17A (CTLA8)	0.1	2.44-10000
Interleukin -18	IL-18	0.4	9.77-40000
Interleukin -21	IL-21	0.6	9.77-40000
Interleukin -22	IL-22	8.2	31.74-130000
Interleukin -23	IL-23	0.9	14.65-60000
Interleukin -27	IL-27	5.1	24.41-100000
Interleukin -31	IL-31	3.3	19.53-80000
Interferon gamma-induced protein-10	IP-10 (CXCL10)	0.3	1.95-8000
Monocyte chemoattractant protein-1	MCP-1 (CCL2)	0.6	1.22-5000
Macrophage inflammatory protein-1 alpha	MIP-1α (CCL3)	1.1	2.14-8750
Macrophage inflammatory protein-1 beta	MIP-1β (CCL4)	4.7	6.10-25000
Regulated on activation, normal T-expressed and secreted	RANTES (CCL5)	0.2	0.61-2500
Stromal cell-derived factor 1 alpha	SDF-1α	20.5	17.09-70000
Tumor necrosis factor alpha	TNF-α	0.4	8.54-35000
Tumor necrosis factor beta	TNF-β	1.6	6.10-25000

Supplementary Table 2: Annotation of the keys used by the Bruker IVDr Lipoproteins Subclass Analysis (B.I.LISA ™)

Abbreviation	Class / Subclass	Compound	Unit
TPTG	Total Plasma	Triglycerides	mg/dL
TPCH	Total Plasma	Cholesterol	mg/dL
LDCH	LDL	Cholesterol	mg/dL
HDCH	HDL	Cholesterol	mg/dL
TPA1	Total Plasma	Apolipoprotein-A1	mg/dL
TPA2	Total Plasma	Apolipoprotein-A2	mg/dL
TPAB	Total Plasma	Apolipoprotein-B100	mg/dL
LDHD	Ratio LDL and HDL Cholesterol	LDL Cholesterol / HDL Cholesterol	-/-
ABA1	Ratio of Apolipoproteins B100 and A1	Apolipoprotein-B100 / Apolipoprotein-A1	-/-
TBPN	Apolipoprotein-B100 carrying particles	Particle Number	nmol/L
VLPN	VLDL	Particle Number	nmol/L
IDPN	IDL	Particle Number	nmol/L
LDPN	LDL	Particle Number	nmol/L
L1PN	LDL-1	Particle Number	nmol/L
L2PN	LDL-2	Particle Number	nmol/L
L3PN	LDL-3	Particle Number	nmol/L
L4PN	LDL-4	Particle Number	nmol/L
L5PN	LDL-5	Particle Number	nmol/L
L6PN	LDL-6	Particle Number	nmol/L
VLTG	VLDL Class	Triglycerides	mg/dL
IDTG	IDL Class	Triglycerides	mg/dL
LDTG	LDL Class	Triglycerides	mg/dL
HDTG	HDL Class	Triglycerides	mg/dL
VLCH	VLDL Class	Cholesterol	mg/dL
IDCH	IDL Class	Cholesterol	mg/dL
VLFC	VLDL Class	Free Cholesterol	mg/dL
IDFC	IDL Class	Free Cholesterol	mg/dL
LDFC	LDL Class	Free Cholesterol	mg/dL
HDFC	HDL Class	Free Cholesterol	mg/dL
VLPL	VLDL Class	Phospholipids	mg/dL
IDPL	IDL Class	Phospholipids	mg/dL
LDPL	LDL Class	Phospholipids	mg/dL
HDPL	HDL Class	Phospholipids	mg/dL
HDA1	HDL Class	Apolipoprotein-A1	mg/dL

HDA2	HDL Class	Apolipoprotein-A2	mg/dL
VLAB	VLDL Class	Apolipoprotein-B100	mg/dL
IDAB	IDL Class	Apolipoprotein-B100	mg/dL
LDAB	LDL Class	Apolipoprotein-B100	mg/dL
V1TG	VLDL-1 Subclass	Triglycerides	mg/dL
V2TG	VLDL-2 Subclass	Triglycerides	mg/dL
V3TG	VLDL-3 Subclass	Triglycerides	mg/dL
V4TG	VLDL-4 Subclass	Triglycerides	mg/dL
V5TG	VLDL-5 Subclass	Triglycerides	mg/dL
V1CH	VLDL-1 Subclass	Cholesterol	mg/dL
V2CH	VLDL-2 Subclass	Cholesterol	mg/dL
V3CH	VLDL-3 Subclass	Cholesterol	mg/dL
V4CH	VLDL-4 Subclass	Cholesterol	mg/dL
V5CH	VLDL-5 Subclass	Cholesterol	mg/dL
V1FC	VLDL-1 Subclass	Free Cholesterol	mg/dL
V2FC	VLDL-2 Subclass	Free Cholesterol	mg/dL
V3FC	VLDL-3 Subclass	Free Cholesterol	mg/dL
V4FC	VLDL-4 Subclass	Free Cholesterol	mg/dL
V5FC	VLDL-5 Subclass	Free Cholesterol	mg/dL
V1PL	VLDL-1 Subclass	Phospholipids	mg/dL
V2PL	VLDL-2 Subclass	Phospholipids	mg/dL
V3PL	VLDL-3 Subclass	Phospholipids	mg/dL
V4PL	VLDL-4 Subclass	Phospholipids	mg/dL
V5PL	VLDL-5 Subclass	Phospholipids	mg/dL
L1TG	LDL-1 Subclass	Triglycerides	mg/dL
L2TG	LDL-2 Subclass	Triglycerides	mg/dL
L3TG	LDL-3 Subclass	Triglycerides	mg/dL
L4TG	LDL-4 Subclass	Triglycerides	mg/dL
L5TG	LDL-5 Subclass	Triglycerides	mg/dL
L6TG	LDL-6 Subclass	Triglycerides	mg/dL
L1CH	LDL-1 Subclass	Cholesterol	mg/dL
L2CH	LDL-2 Subclass	Cholesterol	mg/dL
L3CH	LDL-3 Subclass	Cholesterol	mg/dL
L4CH	LDL-4 Subclass	Cholesterol	mg/dL
L5CH	LDL-5 Subclass	Cholesterol	mg/dL
L6CH	LDL-6 Subclass	Cholesterol	mg/dL
L1FC	LDL-1 Subclass	Free Cholesterol	mg/dL
L2FC	LDL-2 Subclass	Free Cholesterol	mg/dL
L3FC	LDL-3 Subclass	Free Cholesterol	mg/dL
L4FC	LDL-4 Subclass	Free Cholesterol	mg/dL
L5FC	LDL-5 Subclass	Free Cholesterol	mg/dL

L6FC	LDL-6 Subclass	Free Cholesterol	mg/dL
L1PL	LDL-1 Subclass	Phospholipids	mg/dL
L2PL	LDL-2 Subclass	Phospholipids	mg/dL
L3PL	LDL-3 Subclass	Phospholipids	mg/dL
L4PL	LDL-4 Subclass	Phospholipids	mg/dL
L5PL	LDL-5 Subclass	Phospholipids	mg/dL
L6PL	LDL-6 Subclass	Phospholipids	mg/dL
L1AB	LDL-1 Subclass	Apolipoprotein-B100	mg/dL
L2AB	LDL-2 Subclass	Apolipoprotein-B100	mg/dL
L3AB	LDL-3 Subclass	Apolipoprotein-B100	mg/dL
L4AB	LDL-4 Subclass	Apolipoprotein-B100	mg/dL
L5AB	LDL-5 Subclass	Apolipoprotein-B100	mg/dL
L6AB	LDL-6 Subclass	Apolipoprotein-B100	mg/dL
H1TG	HDL-1 Subclass	Triglycerides	mg/dL
H2TG	HDL-2 Subclass	Triglycerides	mg/dL
H3TG	HDL-3 Subclass	Triglycerides	mg/dL
H4TG	HDL-4 Subclass	Triglycerides	mg/dL
H1CH	HDL-1 Subclass	Cholesterol	mg/dL
H2CH	HDL-2 Subclass	Cholesterol	mg/dL
H3CH	HDL-3 Subclass	Cholesterol	mg/dL
H4CH	HDL-4 Subclass	Cholesterol	mg/dL
H1FC	HDL-1 Subclass	Free Cholesterol	mg/dL
H2FC	HDL-2 Subclass	Free Cholesterol	mg/dL
H3FC	HDL-3 Subclass	Free Cholesterol	mg/dL
H4FC	HDL-4 Subclass	Free Cholesterol	mg/dL
H1PL	HDL-1 Subclass	Phospholipids	mg/dL
H2PL	HDL-2 Subclass	Phospholipids	mg/dL
H3PL	HDL-3 Subclass	Phospholipids	mg/dL
H4PL	HDL-4 Subclass	Phospholipids	mg/dL
H1A1	HDL-1 Subclass	Apolipoprotein-A1	mg/dL
H2A1	HDL-2 Subclass	Apolipoprotein-A1	mg/dL
H3A1	HDL-3 Subclass	Apolipoprotein-A1	mg/dL
H4A1	HDL-4 Subclass	Apolipoprotein-A1	mg/dL
H1A2	HDL-1 Subclass	Apolipoprotein-A2	mg/dL
H2A2	HDL-2 Subclass	Apolipoprotein-A2	mg/dL
H3A2	HDL-3 Subclass	Apolipoprotein-A2	mg/dL
H4A2	HDL-4 Subclass	Apolipoprotein-A2	mg/dL

Abbreviations: LDL, low-density lipoprotein; HDL, high-density lipoprotein; VLDL, very low-density lipoprotein; and IDL, intermediate-density lipoprotein.

1. Government A. Australian Technical Advisory Group on Immunisation (ATAGI) _historial statments 2022. Available from: https://www.health.gov.au/committees-and-groups/australian-technical-advisory-group-on-immunisation-atagi#statements.
2. Masuda R, Lodge S, Nitschke P, Spraul M, Schaefer H, Bong SH, et al. Integrative Modeling of Plasma Metabolic and Lipoprotein Biomarkers of SARS-CoV-2 Infection in Spanish and Australian COVID-19 Patient Cohorts. J Proteome Res. 2021;20(8):4139-52. Epub 20210712. doi: 10.1021/acs.jproteome.1c00458. PubMed PMID: 34251833.
3. Lodge S, Nitschke P, Kimhofer T, Coudert JD, Begum S, Bong SH, et al. NMR Spectroscopic Windows on the Systemic Effects of SARS-CoV-2 Infection on Plasma Lipoproteins and Metabolites in Relation to Circulating Cytokines. J Proteome Res. 2021;20:1382.
4. Kimhofer T, Lodge S, Whiley L, Gray N, Loo RL, Lawler NG, et al. Integrative Modeling of Quantitative Plasma Lipoprotein, Metabolic, and Amino Acid Data Reveals a Multiorgan Pathological Signature of SARS-CoV-2 Infection. J Proteome Res. 2020. Epub 2020/09/14. doi: 10.1021/acs.jproteome.0c00519. PubMed PMID: 32806897; PubMed Central PMCID: PMCPMC7489050.
5. Holmes E, Wist J, Masuda R, Lodge S, Nitschke P, Kimhofer T, et al. Incomplete Systemic Recovery and Metabolic Phenoreversion in Post-Acute-Phase Nonhospitalized COVID-19 Patients: Implications for Assessment of Post-Acute COVID-19 Syndrome. J Proteome Res. 2021;20(6):3315-29. Epub 20210519. doi: 10.1021/acs.jproteome.1c00224. PubMed PMID: 34009992; PubMed Central PMCID: PMCPMC8147448.
6. Szczepanek J, Skorupa M, Goroncy A, Jarkiewicz-Tretyn J, Wypych A, Sandomierz D, et al. Anti-SARS-CoV-2 IgG against the S Protein: A Comparison of BNT162b2, mRNA-1273, ChAdOx1 nCoV-2019 and Ad26.COV2.S Vaccines. Vaccines. 2022;10(1). doi: 10.3390/vaccines10010099.
7. Khalifian S, Raimondi G, Brandacher G. The Use of Luminex Assays to Measure Cytokines. Journal of Investigative Dermatology. 2015;135(4):1-5. doi: 10.1038/jid.2015.36.
8. Sanz HA, J. Harezlak, J. Dong, Y. Murawska, M. Valim, C. drLumi: multiplex immunoassays data analysis. 2015.
9. Sanz H, Aponte JJ, Harezlak J, Dong Y, Ayestaran A, Nhabomba A, et al. drLumi: An open-source package to manage data, calibrate, and conduct quality control of multiplex bead-based immunoassays data analysis. PLoS One. 2017;12(11):e0187901. Epub 20171114. doi: 10.1371/journal.pone.0187901. PubMed PMID: 29136653; PubMed Central PMCID: PMCPMC5685631.
10. Dona AC, Jiménez B, Schäfer H, Humpfer E, Spraul M, Lewis MR, et al. Precision high-throughput proton NMR spectroscopy of human urine, serum, and plasma for large-scale metabolic phenotyping. Anal Chem. 2014;86(19):9887-94. Epub 2014/09/16. doi: 10.1021/ac5025039. PubMed PMID: 25180432.
11. Jiménez B, Holmes E, Heude C, Tolson RF, Harvey N, Lodge SL, et al. Quantitative Lipoprotein Subclass and Low Molecular Weight Metabolite Analysis in Human Serum and Plasma by 1H NMR Spectroscopy in a Multilaboratory Trial. Analytical Chemistry. 2018;90(20):11962-71. doi: 10.1021/acs.analchem.8b02412.

12. Loo RL, Lodge S, Kimhofer T, Bong S-H, Begum S, Whiley L, et al. Quantitative In-Vitro Diagnostic NMR Spectroscopy for Lipoprotein and Metabolite Measurements in Plasma and Serum: Recommendations for Analytical Artifact Minimization with Special Reference to COVID-19/SARS-CoV-2 Samples. Journal of Proteome Research. 2020;19(11):4428-41. doi: 10.1021/acs.jproteome.0c00537.
13. Wold S, Esbensen K, Geladi P. Principal Component Analysis. Chemom Intell Lab Syst. 1987;2(1–3):37.
14. Trygg J, Wold S. Orthogonal projections to latent structures (O-PLS). Journal of Chemometrics. 2002;16(3):119-28.
15. Wang JL, Chiou J, Müller HG. Functional data analysis. . Annual Review of Statistics and Its Application 2016;3:257–95. doi: https://doi.org/10.1146/annurev-statistics-041715-033624.
16. Chen K, Zhang X, Petersen A, Müller HG. Quantifying infinite-dimensional data: Functional Data Analysis in action. . Statistics in Biosciences 2017;9:582–-604.
17. Zhou Y, Chen H, Iao S, Kundu P, Zhou H, Bhattacharjee S, et al. fdapace: Functional Data Analysis and Empirical Dynamics 2024 [cited 2024 5/07/2024]. Available from: <https://CRAN.R-project.org/package=fdapace>.
18. Thevenot EA, Roux A, Xu Y, Ezan E, Junot C. Analysis of the Human Adult Urinary Metabolome Variations with Age, Body Mass Index, and Gender by Implementing a Comprehensive Workflow for Univariate and OPLS Statistical Analyses. J Proteome Res. 2015;14(8):3322-35. Epub 20150702. doi: 10.1021/acs.jproteome.5b00354. PubMed PMID: 26088811.
